# Supplementary material for: Postoperative effects of laparoscopic Hartmann reversal: A multicenter propensity score matched study
Source: PLoS One. 2023 Jun 2;18(6):e0286562. doi: 10.1371/journal.pone.0286562 (PMC10237654; doi:10.1371/journal.pone.0286562)
Supplement: Attachment — Submitted filename: PONE-D-23-05434_reviewer.pdf [file pone.0286562.s005.pdf]

PLOS ONE

Postoperative effects of laparoscopic Hartmann reversal: A multicenter propensity score matched study.

--Manuscript Draft--

**Manuscript Number:**	PONE-D-23-05434
**Article Type:**	Research Article
**Full Title:**	Postoperative effects of laparoscopic Hartmann reversal: A multicenter propensity score matched study.
**Short Title:**	Advantage of Laparoscopic Hartmann reversal
**Corresponding Author:**	Jaeim Lee<br>Uijeongbu Saint Mary's Hospital, College of Medicine, The Catholic University of Korea<br>Uijeongbu, KOREA, REPUBLIC OF
**Keywords:**	colon, colostomy, colorectal surgery, laparoscopy
**Abstract:**	Background<br><br>Although the advantages of laparoscopic Hartmann reversal (LHR) compared to open Hartmann reversal (OHR) have been reported in the literature, the number of multicenter studies with good matching investigating this topic is rare. In the present study, we aimed to confirm the advantages of LHR in terms of short-term outcomes through propensity score matching of LHR and OHR groups, using data collected from multiple institutions.<br><br>Methods<br><br>Patients who underwent Hartmann reversal at six institutions under the Catholic Medical Center of the Catholic University of Korea between January 1, 2005, and December 31, 2021, were included. The patients were divided into the LHR and OHR groups based on the technique used. The two groups were matched using propensity score matching (1:1 ratio, logistic regression with the nearest-neighbor method). The primary outcome was postoperative ileus (POI) frequency, and secondary outcomes were time to solid diet (days) and length of stay (days).<br><br>Results<br><br>Among 337 patients, propensity score matching was performed on 322, after excluding 15 who had undergone open conversion. Of these, 63 patients were assigned to each group through propensity score matching. There was no difference in the frequency of adhesiolysis (77.8% vs. 82.5%, p=0.503) or the operation time. (210 (IQR 159-290) vs. 233 (IQR 160-280), p=0.718) between the two groups. As the primary outcome, the LHR group showed significantly lower POI frequency than the OHR group. (4.8% vs. 22.2%, p=0.0041) Regarding the secondary outcomes, the LHR group showed a shorter period to solid diet than the OHR group. The length of hospital stay was also significantly shorter in the LHR group (4 vs. 6, p < 0.0001; 9 vs. 12, p<0.0001).<br><br>Conclusion<br><br>LHR is an effective method to ensure faster recovery of patients after surgery compared to OHR.
**Order of Authors:**	Kil-yong Lee
	Jaeim Lee
	Seong Taek Oh
	Chul Seung Lee
	Nam Suk Kim
	Ju Myung Song

Powered by Editorial Manager® and ProduXion Manager® from Aries Systems Corporation

	Ri-Na Yoo
	Byung Jo Choi
**Additional Information:**	
**Question**	**Response**
**Financial Disclosure**<br><br>Enter a financial disclosure statement that describes the sources of funding for the work included in this submission. Review the submission guidelines for detailed requirements. View published research articles from *PLOS ONE* for specific examples.<br><br>This statement is required for submission and **will appear in the published article** if the submission is accepted. Please make sure it is accurate.<br><br>**Unfunded studies**<br>Enter: *The author(s) received no specific funding for this work.*<br><br>**Funded studies**<br>Enter a statement with the following details:<br>• Initials of the authors who received each award<br>• Grant numbers awarded to each author<br>• The full name of each funder<br>• URL of each funder website<br>• Did the sponsors or funders play any role in the study design, data collection and analysis, decision to publish, or preparation of the manuscript?<br>• **NO** - Include this sentence at the end of your statement: *The funders had no role in study design, data collection and analysis, decision to publish, or preparation of the manuscript.*<br>• **YES** - Specify the role(s) played.<br><br>* typeset	The author(s) received no specific funding for this work.
**Competing Interests**<br><br>Use the instructions below to enter a competing interest statement for this submission. On behalf of all authors, disclose any competing interests that could be perceived to bias this work—acknowledging all financial support and any other relevant financial or non-	The authors have declared that no competing interests exist.

Powered by Editorial Manager® and ProduXion Manager® from Aries Systems Corporation

financial competing interests.<br><br>This statement is required for submission and **will appear in the published article** if the submission is accepted. Please make sure it is accurate and that any funding sources listed in your Funding Information later in the submission form are also declared in your Financial Disclosure statement.<br><br>View published research articles from *PLOS ONE* for specific examples.<br><br>**NO authors have competing interests**<br><br>Enter: *The authors have declared that no competing interests exist.*<br><br>**Authors with competing interests**<br><br>Enter competing interest details beginning with this statement:<br><br>*I have read the journal's policy and the authors of this manuscript have the following competing interests: [insert competing interests here]*<br><br>* typeset	
**Ethics Statement**<br><br>Enter an ethics statement for this submission. This statement is required if the study involved:<br><br>• Human participants<br>• Human specimens or tissue<br>• Vertebrate animals or cephalopods<br>• Vertebrate embryos or tissues<br>• Field research<br><br>Write "N/A" if the submission does not require an ethics statement.<br><br>General guidance is provided below. Consult the submission guidelines for	This study was approved by the institutional review board (IRB) of the Catholic University of Korea and was performed in accordance with the IRB's guidelines and regulations.

detailed instructions. **Make sure that all information entered here is included in the Methods section of the manuscript.**<br><br>**Format for specific study types**<br><br>**Human Subject Research (involving human participants and/or tissue)**<br>• Give the name of the institutional review board or ethics committee that approved the study<br>• Include the approval number and/or a statement indicating approval of this research<br>• Indicate the form of consent obtained (written/oral) or the reason that consent was not obtained (e.g. the data were analyzed anonymously)<br><br>**Animal Research (involving vertebrate animals, embryos or tissues)**<br>• Provide the name of the Institutional Animal Care and Use Committee (IACUC) or other relevant ethics board that reviewed the study protocol, and indicate whether they approved this research or granted a formal waiver of ethical approval<br>• Include an approval number if one was obtained<br>• If the study involved *non-human primates*, add *additional details* about animal welfare and steps taken to ameliorate suffering<br>• If anesthesia, euthanasia, or any kind of animal sacrifice is part of the study, include briefly which substances and/or methods were applied<br><br>**Field Research**<br><br>Include the following details if this study involves the collection of plant, animal, or other materials from a natural setting:<br>• Field permit number<br>• Name of the institution or relevant body that granted permission	
**Data Availability**<br><br>Authors are required to make all data underlying the findings described fully available, without restriction, and from the time of publication. PLOS allows rare	Yes - all data are fully available without restriction

exceptions to address legal and ethical concerns. See the PLOS Data Policy and FAQ for detailed information.<br><br>A Data Availability Statement describing where the data can be found is required at submission. Your answers to this question constitute the Data Availability Statement and **will be published in the article**, if accepted.<br><br>**Important:** Stating 'data available on request from the author' is not sufficient. If your data are only available upon request, select 'No' for the first question and explain your exceptional situation in the text box.<br><br>Do the authors confirm that all data underlying the findings described in their manuscript are fully available without restriction?	
**Describe where the data may be found in full sentences. If you are copying our sample text, replace any instances of XXX with the appropriate details.**<br><br>• If the data are **held or will be held in a public repository**, include URLs, accession numbers or DOIs. If this information will only be available after acceptance, indicate this by ticking the box below. For example: *All XXX files are available from the XXX database (accession number(s) XXX, XXX.).*<br>• If the data are all contained **within the manuscript and/or Supporting Information files**, enter the following: *All relevant data are within the manuscript and its Supporting Information files.*<br>• If neither of these applies but you are able to provide **details of access elsewhere**, with or without limitations, please do so. For example:<br><br>*Data cannot be shared publicly because of [XXX]. Data are available from the XXX Institutional Data Access / Ethics Committee (contact via XXX) for researchers who meet the criteria for*	Data cannot be shared publicly because of our institutional review board(IRB)'s policy. Data are available from the IRB of the Catholic University of Korea (contact via irbujb@catholic.ac.kr) for researchers who meet the criteria for access to confidential data.

Powered by Editorial Manager® and ProduXion Manager® from Aries Systems Corporation

access to confidential data.<br><br>*The data underlying the results presented in the study are available from (include the name of the third party and contact information or URL).*<br>• This text is appropriate if the data are owned by a third party and authors do not have permission to share the data.<br><br>* typeset	
Additional data availability information:	

Click here to access/download;Manuscript;Manuscript_v1.2.docx

Postoperative effects of laparoscopic Hartmann reversal: A multicenter propensity score matched study.

Kil-yong Lee[1], Jaeim Lee[1*], Seong Taek Oh[1], Chul Seung Lee[2], Nam Suk Kim[3], Ju Myung Song[4], Ri-Na Yoo[5], Byung Jo Choi[6]

[1]Department of Surgery, Uijeongbu St. Mary’s Hospital, College of Medicine, The Catholic University of Korea

[2]Department of Surgery, Seoul St. Mary's Hospital, College of Medicine, The Catholic University of Korea

[3]Department of Surgery, Yeouido St. Mary's Hospital, College of Medicine, The Catholic University of Korea

[4]Department of Surgery, Incheon St. Mary’s Hospital, College of Medicine, The Catholic University of Korea

[5]Department of Surgery, St. Vincent’s Hospital, College of Medicine, The Catholic University of Korea

[6]Department of Surgery, Daejeon St. Mary's Hospital, College of Medicine, The Catholic University of Korea

* Corresponding author:

E-mail: lji96@catholic.ac.kr (JIL)

Abstract

Background: Although the advantages of laparoscopic Hartmann reversal (LHR) compared to open Hartmann reversal (OHR) have been reported in the literature, the number of multicenter studies with good matching investigating this topic is rare. In the present study, we aimed to confirm the advantages of LHR in terms of short-term outcomes through propensity score matching of LHR and OHR groups, using data collected from multiple institutions.

Methods: Patients who underwent Hartmann reversal at six institutions under the Catholic Medical Center of the Catholic University of Korea between January 1, 2005, and December 31, 2021, were included. The patients were divided into the LHR and OHR groups based on the technique used. The two groups were matched using propensity score matching (1:1 ratio, logistic regression with the nearest-neighbor method). The primary outcome was postoperative ileus (POI) frequency, and secondary outcomes were time to solid diet (days) and length of stay (days).

Results: Among 337 patients, propensity score matching was performed on 322, after excluding 15 who had undergone open conversion**.** Of these, 63 patients were assigned to each group through propensity score matching. There was no difference in the frequency of adhesiolysis (77.8% vs. 82.5%, p=0.503) or the operation time. (210 (IQR 159-290) vs. 233 (IQR 160-280), p=0.718) between the two groups. As the primary outcome, the LHR group showed significantly lower POI frequency than the OHR group. (4.8% vs. 22.2%, p=0.0041) Regarding the secondary outcomes, the LHR group showed a shorter period to solid diet than the OHR group. The length of hospital stay was also significantly shorter in the LHR group (4 vs. 6, p < 0.0001; 9 vs. 12, p<0.0001).

Conclusion: LHR is an effective method to ensure faster recovery of patients after surgery compared to OHR.

Introduction

Laparoscopic colorectal surgery for colorectal disease has several advantages, including a reduction in both postoperative pain and hospital stay [1-3]. Furthermore, even highly difficult laparoscopic surgeries such as those of adhesive small bowel obstruction can be safely performed [4]. Hartmann's operation is performed when primary anastomosis of the colon is difficult due to severe peritonitis caused by left colonic perforation or unstable vital signs [5], and is often performed in some patients who have undergone Hartmann’s procedure. However, there are cases in which surgeons hesitate to use laparoscopic surgery because of the difficulty of detachment due to peritoneal adhesions caused by a history of previous surgery [6-10]. However, it has been reported that laparoscopic surgery can be performed safely even with single incision during Hartmann reversal [11, 5, 12]. Nevertheless, there is debate regarding the merits of laparoscopic Hartmann’s reversal over open procedures due to the lack of randomized controlled studies.

To address this knowledge gap, in the present study, we aimed to confirm the advantages of laparoscopic Hartmann reversal (LHR) by confirming the difference in postoperative complications using propensity score matching in two groups of patients (laparoscopic vs open approaches) among patients undergoing Hartmann reversal at several centers.

Methods

Patients

Patients who underwent Hartman's reversal at six institutions under the Catholic Medical

Center of the Catholic University of Korea between January 1, 2005 and December 31, 2021, were included. The exclusion criteria were conversion and missing data for the covariates used in propensity score matching. All the data were retrospectively reviewed. This study was approved by the institutional review board (IRB) of the Catholic University of Korea and was performed in accordance with the IRB's guidelines and regulations. The requirement for informed consent was waived by the IRB.

Definition

Postoperative ileus (POI) was defined as any situation that requires a return to "nil per os" or the insertion of a nasogastric tube (NG) [13]. Stump length was measured using sigmoidoscopy prior to surgery. Regarding incisional hernia, defined as a hernia comprising a protruding mass observed from the surgical scar during the follow-up period and confirmed by CT, only patients who underwent CT during the follow-up period to check for incisional hernia were included. Anastomotic stricture was defined as the inability to pass a 13.2-mm colonoscope through the colon, or the feeling of resistance during its passage [14].

Outcomes

The primary outcome was POI frequency, and the secondary outcomes were time to solid diet (days), length of hospital stay (days), and postoperative complications.

Statistical analysis

Patients were divided into two groups: the LHR and the open Hartmann reversal (OHR) groups for comparison of the clinical characteristics. Continuous variables were assessed using the independent t-test or Wilcoxon rank sum test, and discontinuous variables were analyzed using the Chi-square test and Fisher exact test.

Propensity score matching (1:1 ratio using logistic regression with the nearest-neighbor method) was applied to correct for factors that differed between the two groups. The covariates were age, sex, body mass index, smoking, diabetes, hypertension, heart disease, pulmonary disease, liver disease, cerebrovascular disease, stump length, cause of perforation (cancer or benign), combined resection, anastomosis method, and stapler size.

Statistical analyses were performed using SAS ver 9.4 (SAS Institute Inc., Cary, NC, USA). Statistical significance was set at $p<0.05$.

Results

Of the 337 patients who underwent Hartmann’s reversal, 322 were included in this study, after excluding 15 who underwent conversion. Among the 322 included patients, 89 underwent LHR and 233 underwent OHR (Fig 1). The baseline characteristics are shown in Table 1. After propensity score matching, 63 participants were assigned to each group; their baseline characteristics are shown in Table 2.

Fig. 1 Flow diagram of patient selection

Table 1. Baseline characteristics before Propensity Score Matching

		**Baseline characteristics before Propensity Score Matching**		
		**LHR (n=89)**	**OHR (n=233)**	**p-value**
**Age (years)**		69 (IQR 59-75)	66.5 (IQR 57-74)	0.2300(W)
**Sex**	Male	45 (50.56%)	112 (48.07%)	0.6890(C)
	Female	44 (49.44%)	121 (51.93%)	
**Height (cm)**		160 (IQR 151.2-165.2)	157.6 (IQR 151.7-165.0)	0.7861(W)
**Weight (cm)**		58 (IQR 50.6-65.3)	58.7 (IQR 52.0-65.6)	0.8561(W)
**Body mass index (kg/m^2)**		23.2 (IQR 20.9-25.8)	23.4 (IQR 21.2-25.5)	0.8027(W)
**Smoking**	No	76 (85.39%)	192 (82.40%)	0.6819(C)
	Past	5 (5.62%)	12 (5.15%)	
	Present	8 (8.99%)	29 (12.45%)	

**ASA classification**	1	9 (10.23%)	33 (14.16%)	0.6119(C)
	2	68 (77.27%)	169 (72.53%)	
	3	11 (12.50%)	31 (13.30%)	
**Diabetes**	Yes	16 (17.98%)	45 (19.31%)	0.7844(C)
	No	73 (82.02%)	188 (80.69%)	
**Hypertension**	Yes	54 (60.67%)	127 (54.51%)	0.3185(C)
	No	35 (39.33%)	106 (45.49%)	
**Heart disease**	Yes	16 (17.98%)	31 (13.30%)	0.2882(C)
	No	73 (82.02%)	202 (86.70%)	
**Pulmonary disease**	Yes	12 (13.48%)	20 (8.58%)	0.1888(C)
	No	77 (86.52%)	213 (91.42%)	
**Liver disease**	Yes	3 (3.37%)	6 (2.58%)	0.7113(F)
	No	86 (96.63%)	227 (97.42%)	
**Cerebrovascular disease**	Yes	10 (11.24%)	11 (4.72%)	0.0342*(C)
	No	79 (88.76%)	222 (95.28%)	
**Cancer or benign**	Cancer	31 (34.83%)	80 (34.33%)	0.9332(C)
	benign	58 (65.17%)	153 (65.67%)	
**Hartmann method**	Open	19 (21.35%)	202 (87.07%)	<0.0001*(C)
	Laparoscopy	66 (74.16%)	9 (3.88%)	
	Conversion	4 (4.49%)	21 (9.05%)	

LHR, Laparoscopic Hartmann Reversal; OHR, Open Hartmann Reversal; ASA, American Society of Anesthesiologists; IQR, interquartile range

p-value: Independent t-test (T) or Wilcoxon rank sum test (W)

p-value: Chi-square test (C) or Fishers exact test (F)

Table 2. Baseline characteristics after Propensity Score Matching

		**Baseline characteristics after Propensity Score Matching**		
		**LHR (n=63)**	**OHR (n=63)**	**p-value**
**Age (years)**		70 (IQR 62-75)	67 (IQR 57-75)	0.5695(W)
**Sex**	Male	30 (47.62%)	28 (44.44%)	0.7207(C)
	Female	33 (52.38%)	35 (55.56%)	
**Height (cm)**		158 (IQR 150.1-165.0)	156 (IQR 149.7-165.0)	0.8224(W)
**Weight (cm)**		58.73 ± 12.54	58.88 ± 10.83	0.9442(T)
**Body mass index (kg/m^2)**		23.3 (IQR 20.9-25.8)	23.4 (IQR 21.6-24.7)	0.9455(W)
**Smoking**	No	57 (90.48%)	58 (92.06%)	0.5784(F)
	Past	2 (3.17%)	0 (0.00%)	
	Present	4 (6.35%)	5 (7.94%)	
**ASA classification**	1	7 (11.11%)	7 (11.11%)	0.9688(C)

	2	47 (74.60%)	46 (73.02%)	
	3	9 (14.29%)	10 (15.87%)	
**Diabetes**	Yes	10 (15.87%)	10 (15.87%)	1.0000(C)
	No	53 (84.13%)	53 (84.13%)	
**Hypertension**	Yes	39 (61.90%)	41 (65.08%)	0.7113(C)
	No	24 (38.10%)	22 (34.92%)	
**Heart disease**	Yes	12 (19.05%)	9 (14.29%)	0.4733(C)
	No	51 (80.95%)	54 (85.71%)	
**Pulmonary disease**	Yes	6 (9.52%)	4 (6.35%)	0.5098(C)
	No	57 (90.48%)	59 (93.65%)	
**Liver disease**	Yes	1 (1.59%)	1 (1.59%)	1.0000(F)
	No	62 (98.41%)	62 (98.41%)	
**Cerebrovascular disease**	Yes	7 (11.11%)	2 (3.17%)	0.1638(F)
	No	56 (88.89%)	61 (96.83%)	
**Cancer or benign**	Cancer	22 (34.92%)	18 (28.57%)	0.4440(C)
	benign	41 (65.08%)	45 (71.43%)	

LHR, Laparoscopic Hartmann Reversal; OHR, Open Hartmann Reversal; ASA, American Society of Anesthesiologists; IQR, interquartile range

p-value: Independent t-test (T) or Wilcoxon rank sum test (W)

p-value: Chi-square test (C) or Fishers exact test (F)

Operation Related Factors

There were no differences in terms of stump length (p=0.925), adhesiolysis (p=0.503), anastomotic method (p=0.803), diversion (p=0.492), or surgery time (p=0.718) between the two groups (Table 3).

Table 3. Operation related factors

Variable		**Operation related factors**		
		**LHR (n=63)**	**OHR (n=63)**	**p-value**
**Stump length (cm)**		15 (IQR 10-20)	15 (IQR 10-20)	0.9254(W)
**Stump resection**	Yes	11 (17.46%)	12 (19.35%)	0.7846(C)
	No	52 (82.54%)	50 (80.65%)	
**Adhesiolysis**	Yes	49 (77.78%)	52 (82.54%)	0.5028(C)

	No	14 (22.22%)	11 (17.46%)	
**Combined resection**	Yes	9 (14.29%)	10 (15.87%)	0.8034(C)
	No	54 (85.71%)	53 (84.13%)	
**Distance from anal verge to anastomosis (cm)**		11 (IQR 8-15)	15 (IQR 9-17)	0.4869(W)
**Anastomotic method**	end-to-end	54 (85.71%)	53 (84.13%)	0.8034(C)
	Other	9 (14.29%)	10 (15.87%)	
**Circular stapler size (mm)**		28 (IQR 25-28)	28 (IQR 25-28)	0.1771(W)
**Diversion**	Yes	3 (4.76%)	6 (9.52%)	0.4915(F)
	No	60 (95.24%)	57 (90.48%)	
**Surgery time (min)**		210 (IQR 159-290)	233 (IQR 160-280)	0.7180(W)

LHR, Laparoscopic Hartmann Reversal; OHR, Open Hartmann Reversal; IQR, interquartile range

p-value: Independent t-test (T) or Wilcoxon rank sum test (W)

p-value: Chi-square test (C) or Fishers exact test (F)

Primary Outcome

The POI frequencies in the LHR and OHR groups were 4.76% and 22.2%, respectively, showing significant differences (p=0.004) (Table 4).

Table 4. Postoperative outcomes

		Postoperative outcomes		
		**LHR (n=63)**	**OHR (n=63)**	**p-value**
**Length of stay (days)**		9 (IQR 8-11)	12 (IQR 10-16)	**<0.0001*(W)**
**Time to solid diet (days)**		4 (IQR 3-5)	6 (IQR 5-8)	**<0.0001*(W)**
**Clavien-Dindo classification**	<IIIa	60 (95.24%)	58 (92.06%)	0.7175(F)
	≧IIIa	3 (4.76%)	5 (7.94%)	
**Postoperative ileus**	Yes	3 (4.76%)	14 (22.22%)	**0.0041*(C)**
	No	60 (95.24%)	49 (77.78%)	
**Wound infection**	Yes	5 (7.94%)	9 (14.29%)	0.2568(C)

	No	58 (92.06%)	54 (85.71%)	
**Anastomotic stricture**	Yes	0 (0.00%)	2 (7.69%)	0.2189(F)
	No	29 (100.00%)	24 (92.31%)	
**Anastomotic leakage**	Yes	0 (0.00%)	0 (0.00%)	NA
	No	63 (100.00%)	63 (100.00%)	
**Intraabdominal abscess**	Yes	0 (0.00%)	0 (0.00%)	NA
	No	63 (100.00%)	63 (100.00%)	
**Incisional hernia**	Yes	11 (28.95%)	6 (15.38%)	0.1514(C)
	No	27 (71.05%)	33 (84.62%)	

LHR, Laparoscopic Hartmann Reversal; OHR, Open Hartmann Reversal

p-value: Independent t-test (T) or Wilcoxon rank sum test (W)

p-value: Chi-square test (C) or Fishers exact test (F)

Secondary Outcomes

The lengths of stay in the LHR and OHR groups were 9 and 12 days, respectively, showing a significantly shorter length of stay in the LHR group (p<0.001). In terms of time to solid diet, the LHR group showed a significantly shorter period than the OHR group (4 vs 6 days, p<0.001). There was no difference in postoperative complications between the two groups. During the median follow-up period of 346 days, there was no difference in the frequency of incisional hernia between the two groups (p=0.151) (Table 4).

Subgroup analysis

Of the 246 patients who underwent open or conversion Hartmann's operation in included 322 patients (Fig 1), 223 had undergone OHR and 23 had undergone LHR; the frequency of POI between the two groups was 4.4% for LHR and 21.5% for OHR (p=0.055). In terms of time to solid diet, the LHR group (5 days) showed a significantly faster diet than the OHR group (6 days) (p<0.001). The lengths of stay in the LHR and OHR groups were 9 and 12 days, respectively, showing a significantly shorter length of stay in the LHR group (p<0.001). Complications did not differ between the two groups (Supplement Table).

Discussion

Overall, the results of this study showed that LHR reduced POI frequency and decreased the time to solid diet and hospital stay compared to OHR. Similar results were observed in a group of patients who had previously undergone open Hartmann's operation in the subgroup analysis.

In colorectal surgery, the advantages of minimally invasive surgery, such as laparoscopic surgery, compared to open surgery include a more rapid recovery and fewer postoperative complications, such as wound infection. As such, minimally invasive surgery is strongly recommended by the 2018 ERAS Society [15]. However, LHR is more difficult than general laparoscopic surgery because of the presence of intra-abdominal adhesions caused by a past surgical history [6-10]. Nevertheless, the current literature on laparoscopic Hartmann’s reversal highlights the advantages of the laparoscopic approach, such as reducing major complications after surgery and lowering the anastomotic leakage rate [16].

POI is a complication that not only causes patient dissatisfaction, but also prolongs hospital stay [17]. Furthermore, randomized controlled trials using scintigraphy and radiological transit studies to investigate the effect of laparoscopic colorectal surgery on gastrointestinal function confirmed that gastrointestinal function was improved following this surgery [18-20]. LHR has also been reported to significantly reduce POI [21, 22]. Although these studies have the disadvantage of using single-center data, we once again confirmed that LHR plays an important role in reducing POI through propensity score matching using multi-center data.

In our study, LHR shortened both the time to soft diet initiation and the length of hospital stay. This finding agrees with that of a recent meta-analysis [16]. In addition, the recently revised guidelines from the American Society of Colon and Rectal Surgeons and the Society of American Gastrointestinal and Endoscopic Surgeons strongly recommend adopting a

minimally invasive surgical approach because of its advantages in terms of bowel function and length of hospital stay during colorectal surgery [23]. Although LHR is more difficult to perform than general colorectal surgery, if surgeons with the requisite expertise are available, LHR should be performed as it is thought to be helpful in the early recovery of patients, such as speeding up time to solid diet, reducing postoperative pain, and shortening the length of hospital stay.

LHR is a challenging operation because of adhesions due to previous surgical history [6-10]. Accordingly, there are inevitable cases in which it is difficult to complete surgery with the laparoscopic approach. According to a meta-analysis, the average conversion rate is generally around 16.1%, but has been reported to reach up to 50% [24]. In our study, of a total of 104 patients who underwent the laparoscopic approach, 15 (14.4%) required conversion, showing results similar to those in the previous literature.

A recent meta-analysis reported that the frequency of incisional hernia was significantly lower in LHR than that in OHR (OR = 0.42, 95% CI [0.23–0.74], $p < 0.003$) [16]. However, in our study, we observed no difference in the frequency of incisional hernias between the two groups. This may be because only 38 of 63 patients (60.3%) in the LHR group and 39 of 63 patients (61.9%) in the OHR group underwent CT during the follow-up period. It seems necessary to confirm the difference in the frequency of incisional hernia through a multicenter study, including a long-term follow-up.

The primary strength of our study is that it was a multicenter propensity score-matched study. Although this was a retrospective study, post hoc power analysis using the observed differences in the primary outcome variable showed that the result was significant; moreover, the estimated power was confirmed to be approximately 82.6%. However, the present study had some limitations, including the inevitable inclusion of bias owing to the retrospective design. Second,

there was a lack of description regarding the degree of adhesion in the records. Additionally, although the record was not shown, it is possible that selective bias occurred as laparoscopic Hartmann's operation was performed more often in the case of LHR. However, even in the subgroup analysis of the patient group who underwent Open Hartmann's operation, the advantages of LHR were similar to the study results of the entire patient group; therefore, our results support the current recommendations to proceed with the laparoscopic approach if possible.

Conclusion

In conclusion, this study showed that LHR can be performed safely, and has the advantages of reducing POI and length of hospital stay compared with OHR. Because of the limitations of this retrospective study, a multicenter randomized study with long-term follow-up is needed to verify our results.

Acknowledgements

It was supported by Uijeongbu St. Mary's Hospital Clinical Research Coordinating Center as part of the clinical trial activation project.

References

1. Bonjer HJ, Haglind E, Jeekel I, Kazemier G, Pahlman L, Hop WCJ et al. Laparoscopic surgery versus open surgery for colon cancer: short-term outcomes of a randomised trial. Lancet Oncology. 2005;6(7):477-84. doi:10.1016/S1470-2045(05)70221-7.

2. Guillou PJ, Quirke P, Thorpe H, Walker J, Jayne DG, Smith AM et al. Short-term endpoints

of conventional versus laparoscopic-assisted surgery in patients with colorectal cancer (MRC CLASICC trial): multicentre, randomised controlled trial. Lancet. 2005;365(9472):1718-26. doi:10.1016/S0140-6736(05)66545-2.

3. Leung KL, Kwok SP, Lam SC, Lee JF, Yiu RY, Ng SS et al. Laparoscopic resection of rectosigmoid carcinoma: prospective randomised trial. Lancet. 2004;363(9416):1187-92. doi:10.1016/S0140-6736(04)15947-3.

4. Sallinen V, Di Saverio S, Haukijarvi E, Juusela R, Wikstrom H, Koivukangas V et al. Laparoscopic versus open adhesiolysis for adhesive small bowel obstruction (LASSO): an international, multicentre, randomised, open-label trial. Lancet Gastroenterol Hepatol. 2019;4(4):278-86. doi:10.1016/S2468-1253(19)30016-0.

5. Gachabayov M, Oberkofler CE, Tuech JJ, Hahnloser D, Bergamaschi R. Resection with primary anastomosis vs nonrestorative resection for perforated diverticulitis with peritonitis: a systematic review and meta-analysis. Colorectal Dis. 2018;20(9):753-70. doi:10.1111/codi.14237.

6. Banerjee S, Leather AJM, Rennie JA, Samano N, Gonzalez JG, Papagrigoriadis S. Feasibility and morbidity of reversal of Hartmann's. Colorectal Disease. 2005;7(5):454-9. doi:10.1111/j.1463-1318.2005.00862.x.

7. Cellini C, Deeb AP, Sharma A, Monson JRT, Fleming FJ. Association between operative approach and complications in patients undergoing Hartmann's reversal. British Journal of Surgery. 2013;100(8):1094-9. doi:10.1002/bjs.9153.

8. Garber A, Hyman N, Osler T. Complications of Hartmann takedown in a decade of preferred primary anastomosis. American Journal of Surgery. 2014;207(1):60-4. doi:10.1016/j.amjsurg.2013.05.006.

9. Hess GF, Schafer J, Rosenthal R, Kettelhack C, Oertli D. Reversal after Hartmann's procedure in patients with complicated sigmoid diverticulitis. Colorectal Disease.

2017;19(6):582-8. doi:10.1111/codi.13553.

10. Hodgson R, An V, Stupart DA, Guest GD, Watters DAK. Who gets Hartmann's reversed in a regional centre? Surgeon-Journal of the Royal Colleges of Surgeons of Edinburgh and Ireland. 2016;14(4):184-9. doi:10.1016/j.surge.2014.11.001.

11. Clermonts SH, de Ruijter WM, van Loon YT, Wasowicz DK, Heisterkamp J, Maring JK et al. Reversal of Hartmann's procedure utilizing single-port laparoscopy: an attractive alternative to laparotomy. Surg Endosc. 2016;30(5):1894-901. doi:10.1007/s00464-015-4407-3.

12. Thambi P, Borowski DW, Sathasivam R, Obuobi RB, Viswanath YKS, Gill TS. Single-incision laparoscopic reversal of Hartmann's operation through the stoma site: comparative outcomes with conventional laparoscopic and open surgery. Colorectal Disease. 2019;21(7):833-40. doi:10.1111/codi.14617.

13. Delaney CP, Senagore AJ, Gerkin TM, Beard TL, Zingaro WM, Tomaszewski KJ et al. Association of surgical care practices with length of stay and use of clinical protocols after elective bowel resection: results of a national survey. Am J Surg. 2010;199(3):299-304. doi:10.1016/j.amjsurg.2009.08.027.

14. Kee HH, Sik JC, Hee LD, Cheol KH, Sik YC, Kyu PS et al. Anastomotic Stricture after Colorectal Stapled Anastomosis. J Korean Soc Coloproctol. 2000;16(3):198-203.

15. Gustafsson UO, Scott MJ, Hubner M, Nygren J, Demartines N, Francis N et al. Guidelines for Perioperative Care in Elective Colorectal Surgery: Enhanced Recovery After Surgery (ERAS((R))) Society Recommendations: 2018. World J Surg. 2019;43(3):659-95. doi:10.1007/s00268-018-4844-y.

16. Chavrier D, Alves A, Menahem B. Is laparoscopy a reliable alternative to laparotomy in Hartmann's reversal? An updated meta-analysis. Techniques in Coloproctology. 2022;26(4):239-52. doi:10.1007/s10151-021-02560-2.

17. Stein SL. Perioperative management. Clin Colon Rectal Surg. 2013;26(3):137-8.

doi:10.1055/s-0033-1351141.

18. Basse L, Madsen JL, Billesbolle P, Bardram L, Kehlet H. Gastrointestinal transit after laparoscopic versus open colonic resection. Surg Endosc. 2003;17(12):1919-22. doi:10.1007/s00464-003-9013-0.

19. Schwenk W, Bohm B, Haase O, Junghans T, Muller JM. Laparoscopic versus conventional colorectal resection: a prospective randomised study of postoperative ileus and early postoperative feeding. Langenbecks Arch Surg. 1998;383(1):49-55. doi:10.1007/s004230050091.

20. van Bree SH, Vlug MS, Bemelman WA, Hollmann MW, Ubbink DT, Zwinderman AH et al. Faster recovery of gastrointestinal transit after laparoscopy and fast-track care in patients undergoing colonic surgery. Gastroenterology. 2011;141(3):872-80 e1-4. doi:10.1053/j.gastro.2011.05.034.

21. Ng DC, Guarino S, Yau SL, Fok BK, Cheung HY, Li MK et al. Laparoscopic reversal of Hartmann's procedure: safety and feasibility. Gastroenterol Rep (Oxf). 2013;1(2):149-52. doi:10.1093/gastro/got018.

22. Yang PF, Morgan MJ. Laparoscopic versus open reversal of Hartmann's procedure: a retrospective review. ANZ J Surg. 2014;84(12):965-9. doi:10.1111/ans.12667.

23. Irani JL, Hedrick TL, Miller TE, Lee LWC, Steinhagen E, Shogan BD et al. Clinical practice guidelines for enhanced recovery after colon and rectal surgery from the American Society of Colon and Rectal Surgeons and the Society of American Gastrointestinal and Endoscopic Surgeons. Surg Endosc. 2022. doi:10.1007/s00464-022-09758-x.

24. Celentano V, Giglio MC, Bucci L. Laparoscopic versus open Hartmann's reversal: a systematic review and meta-analysis. Int J Colorectal Dis. 2015;30(12):1603-15. doi:10.1007/s00384-015-2325-4.

Supporting information

S1 Table. Baseline characteristics

S2 Table. Operation related factors

S3 Table. Postoperative outcomes

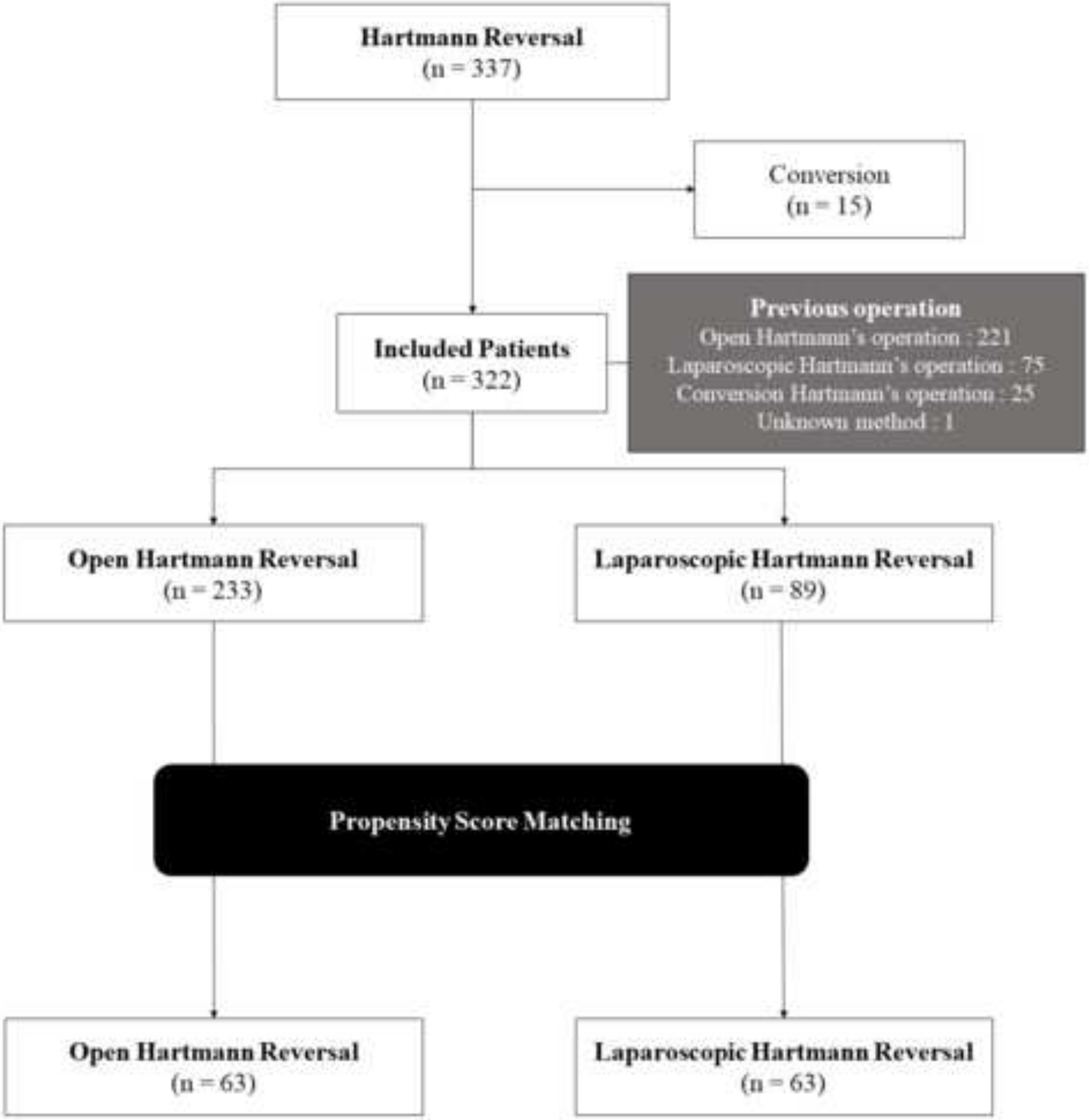
Hartmann Reversal
(n = 337)
Conversion
(n = 15)
Included Patients
(n = 322)
Previous operation
Open Hartmann's operation : 221
Laparoscopic Hartmann's operation : 75
Conversion Hartmann's operation : 25
Unknown method : 1
Open Hartmann Reversal
(n = 233)
Laparoscopic Hartmann Reversal
(n = 89)
Propensity Score Matching
Open Hartmann Reversal
(n = 63)
Laparoscopic Hartmann Reversal
(n = 63)

Click here to access/download
Supporting Information
Supplement Tables.xlsx
