# Supplementary material for: Postoperative effects of laparoscopic Hartmann reversal: A multicenter propensity score matched study
Source: PLoS One. 2023 Jun 2;18(6):e0286562. doi: 10.1371/journal.pone.0286562 (PMC10237654; doi:10.1371/journal.pone.0286562)
Supplement: Attachment — Submitted filename: PONE-D-23-05434_R1.pdf [file pone.0286562.s008.pdf]

PLOS ONE

Postoperative effects of laparoscopic Hartmann reversal: A multicenter propensity score matched study.

--Manuscript Draft--

**Manuscript Number:**	PONE-D-23-05434R1
**Article Type:**	Research Article
**Full Title:**	Postoperative effects of laparoscopic Hartmann reversal: A multicenter propensity score matched study.
**Short Title:**	Advantage of Laparoscopic Hartmann reversal
**Corresponding Author:**	Jaeim Lee<br>Uijeongbu Saint Mary's Hospital, College of Medicine, The Catholic University of Korea<br>Uijeongbu, KOREA, REPUBLIC OF
**Keywords:**	colon, colostomy, colorectal surgery, laparoscopy
**Abstract:**	Background<br><br>Although the advantages of laparoscopic Hartmann reversal (LHR) compared to open Hartmann reversal (OHR) have been reported in the literature, the number of multicenter studies with good matching investigating this topic is rare. In the present study, we aimed to confirm the advantages of LHR in terms of short-term outcomes through propensity score matching of LHR and OHR groups, using data collected from multiple institutions.<br><br>Methods<br><br>Patients who underwent Hartmann reversal at six institutions under the Catholic Medical Center of the Catholic University of Korea between January 1, 2005, and December 31, 2021, were included. The patients were divided into the LHR and OHR groups based on the technique used. The two groups were matched using propensity score matching (1:1 ratio, logistic regression with the nearest-neighbor method). The primary outcome was postoperative ileus (POI) frequency, and secondary outcomes were time to solid diet (days) and length of stay (days).<br><br>Results<br><br>Among 337 patients, propensity score matching was performed on 322, after excluding 15 who had undergone open conversion. Of these, 63 patients were assigned to each group through propensity score matching. There was no difference in the frequency of adhesiolysis (77.8% vs. 82.5%, p=0.503) or the operation time. (210 (IQR 159-290) vs. 233 (IQR 160-280), p=0.718) between the two groups. As the primary outcome, the LHR group showed significantly lower POI frequency than the OHR group. (4.8% vs. 22.2%, p=0.0041) Regarding the secondary outcomes, the LHR group showed a shorter period to solid diet than the OHR group. The length of hospital stay was also significantly shorter in the LHR group (4 vs. 6, p < 0.0001; 9 vs. 12, p<0.0001).<br><br>Conclusion<br><br>LHR is an effective method to ensure faster recovery of patients after surgery compared to OHR.
**Order of Authors:**	Kil-yong Lee
	Jaeim Lee
	Seong Taek Oh
	Chul Seung Lee
	Nam Suk Kim
	Ju Myung Song

Powered by Editorial Manager® and ProduXion Manager® from Aries Systems Corporation

	Ri-Na Yoo
	Byung Jo Choi
**Response to Reviewers:**	Review Comments to the Author<br><br>Reviewer #1: Title: Postoperative effects of laparoscopic Hartmann reversal: A multicenter propensity<br>score matched study.<br>The title is in line with study objectives and the results<br>Abstract: Structured and well written<br>Background: Detailed information on statement of problem as well as rational for the study clearly presented.<br>Methods: Fairly well described.<br>• Comment: Study design should be mentioned even though it is apparent from the methodology.<br><br>We thank the reviewer for the considerate comments. Accordingly, we have indicated the study design at the beginning of the methods.<br><br>• Comment: Minimum sample size for the study was not calculated. This is desirable, as this will show whether the sample size in this study meets the minimum calculated sample size. If the sample size used is less than the calculated minimum sample size, then this can be a limitation in the study.<br>According to the reviewer's comments, we have calculated sample size. As reported by Ng et al. [1] and Yang et al. [2], with 80% power and alpha 0.05, the sample size required for each group was 60 patients. Therefore, in this study, the number of samples included is appropriate, with a total of 126 patients and 63 patients in each group; the power was confirmed to be about 82% in the power analysis. This information has been added it in the "Patients" and "Results" section.<br>References<br>1. Ng DC, Guarino S, Yau SL, Fok BK, Cheung HY, Li MK et al. Laparoscopic reversal of Hartmann's procedure: safety and feasibility. Gastroenterol Rep (Oxf). 2013;1(2):149-52. doi:10.1093/gastro/got018.<br>2. Yang PF, Morgan MJ. Laparoscopic versus open reversal of Hartmann's procedure: a retrospective review. ANZ J Surg. 2014;84(12):965-9. doi:10.1111/ans.12667.<br><br>Result: Well written in details with relevant tables and figures<br>• Comment: The first column for most of the tables are without a label<br>Accordingly, we have added the label to the first column.<br><br>• Comment: The state number of days for POI, hospital stay, etc are not really clear. Are these mean values or median? If it is mean value, please indicate it in the sentence and provide the standard deviation (SD) as well.<br>The number of days for hospital stay and the time to solid diet are described as the median. These have been added to the results section.<br><br>• Comment: Line 126 - Frequency cannot be in percentage. Please correct it. Is it the prevalence?<br>This indicates the prevalence of POIs in each group. According to the reviewer's comment, we have changed it to the number of occurrences as well as the percentage.<br><br>Discussion: The study findings are well discussed, with study limitations provided.<br>Conclusion: Clearly written with appropriate recommendation.<br><br>Reviewer #2: The author focused to the short-term outcomes of reversal Hartmann operation and compared the advantages of laparoscopic and open surgery in this operation. They enrolled the patients from multi-institutions retrospectively and used the propensity score matched analysis for deep statistical analysis. From the analysis, the author have concluded that laparoscopic Hartmann reversal (LHR) is better method for fast recovery from the surgery and to prevent the postoprative bowel obstrution than open Hartmann reversal (OHR). Overall, the analysis seems to be fair and the results of this manuscript are similar to the previous studies and I agree with it. As the author pointed out, the study which reported the outcome of Hartmann reversal operation is

not that many and these previous publications are mainly from single institution. This presented study is multi-instituional study with propensity score matced analysis and these background seems to strengthen the result compared to the previous studies. However, there are several concerns in this study which I want to point out.

1. Comment: As the author pointed out, the peritoneal adhesion will be the challenge to perform LHR. I feel that the conversion rate of LHR is another important outcome and I do not understand why the author excluded the 15 conversion cases from the analysis. It is more important to know how the LHR is difficult and conversion to open surgery may happen. Looking into the result, it seems that about 15% of patients were converted to open surgery and I recommend the author to analyze and discuss more deeply about it.
We appreciate the reviewer's considerate comments. The initial intention of the group that underwent conversion was LHR; thus, if it were analyzed by intention-to-treat (ITT) method, this group should be analyzed by putting it in the LHR group. However, because the surgical method itself was open, it was excluded from our study. Nonetheless, according to the reviewer's opinion, we performed further analysis to investigate whether a conversion group differs from other groups. Although the surgical time was longer in this group compared to that in the LHR group, there was no difference in other postoperative complications and POI. We have added a supplement table summarizing the analysis of the three groups and have summarized the results in the discussion section.

2. Comment: In Table 1, I see the data of the previous surgery (Hartmann method) but this data is not presented in Table 2. This is very important information to consider the result of matching and I highly recommend to include it.
In the group that underwent open Hartmann's reversal, remarkably few cases were present where the previous operation was laparoscopic Hartmann's operation (3.9%); thus, we excluded Hartmann's method from matching in PSM and included this as a limitation in the discussion section. However, according to the reviewer's opinion, we have added the Hartmann method to Table 2 as well.

3. Comment: Hartmann operation will be considered when the anastomosis is considered to be difficult. However, reason of this decision will be various kinds of things; for example, perforation, severe comorbidity, elderly patients, proficiency of the surgeon, etc. It would be better to include more specifc data why the patients underwent Hartmann operation at the beginning.
Our study focused on Hartmann's reversal and investigated data from multi-institutional electric medical records. Furthermore, there were cases where Hartmann's operation was performed at institutions other than our six institutions, and there were no records from the other hospital; even though there was a surgical record for Hartmann's operation, there was no mention of the reason why Hartmann's operation was performed, and we had to exclude that item. Therefore, we have added this as a limitation of our study in the discussion section.

4. Comment: As the author mentioned in the limitation, there will be a selection bias since the patients with advanced cancer cannot undergo Hartmann reversal when they are under chemotherapy. It might be better to show how many patients underwent Hartmann surgery and how many had Hartmann reveersal.
According to the reviewer's opinion, the number of patients who underwent Hartmann's surgery in our institutes was investigated. In total, 1076 patients underwent Hartmann's operation in our hospital, and among them, 306 patients underwent Hartmann reversal. The remaining 31 patients who underwent Hartmann reversal in our institutes were patients who had undergone Hartmann's operation at other hospitals. This has been added to Figure 1.

5. Comment: The frequency of POI seems to be quite different. Is there any difference in the required treatment for these patient comparing LHR and OHR group? Were there any case which needed another surgery to treat POI? The primary outcome was set as POI and I recommend the author to analyze more deeply in it.
In the LHR group, two POI patients showed an improvement in symptoms at 2 and 3 days, respectively, after L-tube insertion with parenteral nutritional support. However, one patient underwent laparoscopic adhesiolysis with transverse colectomy to resolve adhesions.

Powered by Editorial Manager® and ProduXion Manager® from Aries Systems Corporation

	Of 13 POI patients in the OHR group, three patients showed spontaneous improvement in their symptoms without L-tube insertion. However, one patient underwent open adhesiolysis with T-colostomy to resolve the adhesion.<br>We have added these in the results section.<br><br>6. Comment: Although there was no significant difference, I am quite surprised that the incisional hernia was more frequent in LHR group. Why do you think this happened?<br>The exact cause is not known, but the occurrence of incisional hernia was confirmed by CT; since CT was performed in only about 38 out of 63 patients in each group, this may have resulted in a difference in incidence rates.
Additional Information:	
Question	Response
**Financial Disclosure**<br><br>Enter a financial disclosure statement that describes the sources of funding for the work included in this submission. Review the submission guidelines for detailed requirements. View published research articles from *PLOS ONE* for specific examples.<br><br>This statement is required for submission and **will appear in the published article** if the submission is accepted. Please make sure it is accurate.<br><br>**Unfunded studies**<br>Enter: *The author(s) received no specific funding for this work.*<br><br>**Funded studies**<br>Enter a statement with the following details:<br>• Initials of the authors who received each award<br>• Grant numbers awarded to each author<br>• The full name of each funder<br>• URL of each funder website<br>• Did the sponsors or funders play any role in the study design, data collection and analysis, decision to publish, or preparation of the manuscript?<br>• **NO** - Include this sentence at the end of your statement: *The funders had no role in study design, data collection and analysis, decision to publish, or preparation of the manuscript.*<br>• **YES** - Specify the role(s) played.<br><br>* typeset	The author(s) received no specific funding for this work.
**Competing Interests**<br><br>Use the instructions below to enter a	The authors have declared that no competing interests exist.

competing interest statement for this submission. On behalf of all authors, disclose any competing interests that could be perceived to bias this work—acknowledging all financial support and any other relevant financial or non-financial competing interests.<br><br>This statement is required for submission and **will appear in the published article** if the submission is accepted. Please make sure it is accurate and that any funding sources listed in your Funding Information later in the submission form are also declared in your Financial Disclosure statement.<br><br>View published research articles from *PLOS ONE* for specific examples.<br><br>**NO authors have competing interests**<br><br>Enter: *The authors have declared that no competing interests exist.*<br><br>**Authors with competing interests**<br><br>Enter competing interest details beginning with this statement:<br><br>*I have read the journal's policy and the authors of this manuscript have the following competing interests: [insert competing interests here]*<br><br>* typeset	
**Ethics Statement**<br><br>Enter an ethics statement for this submission. This statement is required if the study involved:<br><br>• Human participants<br>• Human specimens or tissue<br>• Vertebrate animals or cephalopods<br>• Vertebrate embryos or tissues<br>• Field research	This study was approved by the institutional review board (IRB) of the Catholic University of Korea and was performed in accordance with the IRB’s guidelines and regulations.

Write "N/A" if the submission does not require an ethics statement.

General guidance is provided below. Consult the submission guidelines for detailed instructions. **Make sure that all information entered here is included in the Methods section of the manuscript.**

Format for specific study types

Human Subject Research (involving human participants and/or tissue)

- Give the name of the institutional review board or ethics committee that approved the study
- Include the approval number and/or a statement indicating approval of this research
- Indicate the form of consent obtained (written/oral) or the reason that consent was not obtained (e.g. the data were analyzed anonymously)

Animal Research (involving vertebrate animals, embryos or tissues)

- Provide the name of the Institutional Animal Care and Use Committee (IACUC) or other relevant ethics board that reviewed the study protocol, and indicate whether they approved this research or granted a formal waiver of ethical approval
- Include an approval number if one was obtained
- If the study involved *non-human primates*, add *additional details* about animal welfare and steps taken to ameliorate suffering
- If anesthesia, euthanasia, or any kind of animal sacrifice is part of the study, include briefly which substances and/or methods were applied

Field Research

Include the following details if this study involves the collection of plant, animal, or other materials from a natural setting:

- Field permit number
- Name of the institution or relevant body that granted permission

Powered by Editorial Manager® and ProduXion Manager® from Aries Systems Corporation

**Data Availability**<br><br>Authors are required to make all data underlying the findings described fully available, without restriction, and from the time of publication. PLOS allows rare exceptions to address legal and ethical concerns. See the PLOS Data Policy and FAQ for detailed information.<br><br>A Data Availability Statement describing where the data can be found is required at submission. Your answers to this question constitute the Data Availability Statement and **will be published in the article**, if accepted.<br><br>**Important:** Stating 'data available on request from the author' is not sufficient. If your data are only available upon request, select 'No' for the first question and explain your exceptional situation in the text box.<br><br>Do the authors confirm that all data underlying the findings described in their manuscript are fully available without restriction?	Yes - all data are fully available without restriction
**Describe where the data may be found in full sentences. If you are copying our sample text, replace any instances of XXX with the appropriate details.**<br><br>• If the data are **held or will be held in a public repository**, include URLs, accession numbers or DOIs. If this information will only be available after acceptance, indicate this by ticking the box below. For example: *All XXX files are available from the XXX database (accession number(s) XXX, XXX.)*.<br>• If the data are all contained **within the manuscript and/or Supporting Information files**, enter the following: *All relevant data are within the manuscript and its Supporting Information files.*<br>• If neither of these applies but you are able to provide **details of access elsewhere**, with or without limitations, please do so. For example:	Data cannot be shared publicly because of our institutional review board(IRB)'s policy. Data are available from the IRB of the Catholic University of Korea (contact via irbujb@catholic.ac.kr) for researchers who meet the criteria for access to confidential data.

Data cannot be shared publicly because of [XXX]. Data are available from the XXX Institutional Data Access / Ethics Committee (contact via XXX) for researchers who meet the criteria for access to confidential data.<br><br>*The data underlying the results presented in the study are available from (include the name of the third party and contact information or URL).*<br>• This text is appropriate if the data are owned by a third party and authors do not have permission to share the data.<br><br>* typeset	
Additional data availability information:	

Manuscript Click here to access/download;Manuscript;Manuscript.docx

Postoperative effects of laparoscopic Hartmann reversal: A multicenter propensity score matched study.

Kil-yong Lee[1], Jaeim Lee[1*], Seong Taek Oh[1], Chul Seung Lee[2], Nam Suk Kim[3], Ju Myung Song[4], Ri-Na Yoo[5], Byung Jo Choi[6]

[1]Department of Surgery, Uijeongbu St. Mary’s Hospital, College of Medicine, The Catholic University of Korea

[2]Department of Surgery, Seoul St. Mary's Hospital, College of Medicine, The Catholic University of Korea

[3]Department of Surgery, Yeouido St. Mary's Hospital, College of Medicine, The Catholic University of Korea

[4]Department of Surgery, Incheon St. Mary’s Hospital, College of Medicine, The Catholic University of Korea

[5]Department of Surgery, St. Vincent’s Hospital, College of Medicine, The Catholic University of Korea

[6]Department of Surgery, Daejeon St. Mary's Hospital, College of Medicine, The Catholic University of Korea

* Corresponding author:

E-mail: lji96@catholic.ac.kr (JIL)

Abstract

Background: Although the advantages of laparoscopic Hartmann reversal (LHR) compared to open Hartmann reversal (OHR) have been reported in the literature, the number of multicenter studies with good matching investigating this topic is rare. In the present study, we aimed to confirm the advantages of LHR in terms of short-term outcomes through propensity score matching of LHR and OHR groups, using data collected from multiple institutions.

Methods: Patients who underwent Hartmann reversal at six institutions under the Catholic Medical Center of the Catholic University of Korea between January 1, 2005, and December 31, 2021, were included. The patients were divided into the LHR and OHR groups based on the technique used. The two groups were matched using propensity score matching (1:1 ratio, logistic regression with the nearest-neighbor method). The primary outcome was postoperative ileus (POI) frequency, and secondary outcomes were time to solid diet (days) and length of stay (days).

Results: Among 337 patients, propensity score matching was performed on 322, after excluding 15 who had undergone open conversion**.** Of these, 63 patients were assigned to each group through propensity score matching. There was no difference in the frequency of adhesiolysis (77.8% vs. 82.5%, p=0.503) or the operation time. (210 (IQR 159-290) vs. 233 (IQR 160-280), p=0.718) between the two groups. As the primary outcome, the LHR group showed significantly lower POI frequency than the OHR group. (4.8% vs. 22.2%, p=0.0041) Regarding the secondary outcomes, the LHR group showed a shorter period to solid diet than the OHR group. The length of hospital stay was also significantly shorter in the LHR group (4 vs. 6, p < 0.0001; 9 vs. 12, p<0.0001).

Conclusion: LHR is an effective method to ensure faster recovery of patients after surgery compared to OHR.

Keywords: colon, colostomy, colorectal surgery, laparoscopy

Introduction

Laparoscopic colorectal surgery for colorectal disease has several advantages, including a reduction in both postoperative pain and hospital stay [1-3]. Furthermore, even highly difficult laparoscopic surgeries such as those of adhesive small bowel obstruction can be safely performed [4]. Hartmann's operation is performed when primary anastomosis of the colon is difficult due to severe peritonitis caused by left colonic perforation or unstable vital signs [5], and Hartmann reversal is often performed in some patients who have undergone Hartmann’s procedure. However, there are cases in which surgeons hesitate to use laparoscopic surgery because of the difficulty of detachment due to peritoneal adhesions caused by a history of previous surgery [6-10]. However, it has been reported that laparoscopic surgery can be performed safely even with single incision during Hartmann reversal [11, 5, 12]. Nevertheless, there is debate regarding the merits of laparoscopic Hartmann’s reversal over open procedures due to the lack of randomized controlled studies.

To address this knowledge gap, in the present study, we aimed to confirm the advantages of laparoscopic Hartmann reversal (LHR) by confirming the difference in postoperative complications using propensity score matching in two groups of patients (laparoscopic vs open approaches) among patients undergoing Hartmann reversal at several centers.

Methods

This is a multicenter retrospective cohort study. This study was approved by the institutional review board (IRB) of the Catholic University of Korea and was performed in accordance with the IRB's guidelines and regulations. The requirement for informed consent was waived by the IRB.

Patients

Patients who underwent Hartman's reversal at six institutions under the Catholic Medical Center of the Catholic University of Korea between January 1, 2005 and December 31, 2021, were included. The exclusion criteria were conversion and missing data for the covariates used in propensity score matching. All the data were retrospectively reviewed.

As reported by Ng et al. [13] and Yang et al. [14], when the sample size was calculated with 80% power and alpha 0.05, the minimum sample size required for each group was 60 patients.

Definition

Postoperative ileus (POI) was defined as any situation that requires a return to "nil per os" or the insertion of a nasogastric tube (NG) [15]. Stump length was measured using sigmoidoscopy prior to surgery. Regarding incisional hernia, defined as a hernia comprising a protruding mass observed from the surgical scar during the follow-up period and confirmed by CT, only patients who underwent CT during the follow-up period to check for incisional hernia were included. Anastomotic stricture was defined as the inability to pass a 13.2-mm colonoscope through the colon, or the feeling of resistance during its passage [16].

Outcomes

The primary outcome was POI frequency, and the secondary outcomes were time to solid diet (days), length of hospital stay (days), and postoperative complications.

Statistical analysis

Patients were divided into two groups: the LHR and the open Hartmann reversal (OHR) groups for comparison of the clinical characteristics. Continuous variables were assessed using the independent t-test or Wilcoxon rank sum test, and discontinuous variables were analyzed using the Chi-square test and Fisher exact test.

Propensity score matching (1:1 ratio using logistic regression with the nearest-neighbor method) was applied to correct for factors that differed between the two groups. The covariates were age, sex, body mass index, smoking, diabetes, hypertension, heart disease, pulmonary disease, liver disease, cerebrovascular disease, stump length, cause of perforation (cancer or benign), combined resection, anastomosis method, and stapler size.

Statistical analyses were performed using SAS ver 9.4 (SAS Institute Inc., Cary, NC, USA). Statistical significance was set at $p<0.05$.

Results

Of the 337 patients who underwent Hartmann's reversal, 322 were included in this study, after excluding 15 who underwent conversion. Among the 322 included patients, 89 underwent LHR and 233 underwent OHR (Fig 1). The baseline characteristics are shown in Table 1. After propensity score matching, 63 participants were assigned to each group; their baseline characteristics are shown in Table 2. The post-matching patient number of this study was shown to be 82% in power analysis, which was considered an appropriate sample size.

Fig. 1 Flow diagram of patient selection

Table 1. Baseline characteristics before Propensity Score Matching

	**Baseline characteristics before Propensity Score Matching**

Variables		LHR (n=89)	OHR (n=233)	p-value
**Age (years)**		69 (IQR 59-75)	66.5 (IQR 57-74)	0.2300(W)
**Sex**	Male	45 (50.56%)	112 (48.07%)	0.6890(C)
	Female	44 (49.44%)	121 (51.93%)	
**Height (cm)**		160 (IQR 151.2-165.2)	157.6 (IQR 151.7-165.0)	0.7861(W)
**Weight (kg)**		58 (IQR 50.6-65.3)	58.7 (IQR 52.0-65.6)	0.8561(W)
**Body mass index (kg/m^2)**		23.2 (IQR 20.9-25.8)	23.4 (IQR 21.2-25.5)	0.8027(W)
**Smoking**	No	76 (85.39%)	192 (82.40%)	0.6819(C)
	Past	5 (5.62%)	12 (5.15%)	
	Present	8 (8.99%)	29 (12.45%)	
**ASA classification**	1	9 (10.23%)	33 (14.16%)	0.6119(C)
	2	68 (77.27%)	169 (72.53%)	
	3	11 (12.50%)	31 (13.30%)	
**Diabetes**	Yes	16 (17.98%)	45 (19.31%)	0.7844(C)
	No	73 (82.02%)	188 (80.69%)	
**Hypertension**	Yes	54 (60.67%)	127 (54.51%)	0.3185(C)
	No	35 (39.33%)	106 (45.49%)	
**Heart disease**	Yes	16 (17.98%)	31 (13.30%)	0.2882(C)
	No	73 (82.02%)	202 (86.70%)	
**Pulmonary disease**	Yes	12 (13.48%)	20 (8.58%)	0.1888(C)
	No	77 (86.52%)	213 (91.42%)	
**Liver disease**	Yes	3 (3.37%)	6 (2.58%)	0.7113(F)
	No	86 (96.63%)	227 (97.42%)	
**Cerebrovascular disease**	Yes	10 (11.24%)	11 (4.72%)	0.0342*(C)
	No	79 (88.76%)	222 (95.28%)	
**Cancer or benign**	Cancer	31 (34.83%)	80 (34.33%)	0.9332(C)
	benign	58 (65.17%)	153 (65.67%)	
**Hartmann method**	Open	19 (21.35%)	202 (87.07%)	<0.0001*(C)
	Laparoscopy	66 (74.16%)	9 (3.88%)	
	Conversion	4 (4.49%)	21 (9.05%)	

LHR, Laparoscopic Hartmann Reversal; OHR, Open Hartmann Reversal; ASA, American Society of Anesthesiologists; IQR, interquartile range

p-value: Independent t-test (T) or Wilcoxon rank sum test (W)

p-value: Chi-square test (C) or Fishers exact test (F)

Table 2. Baseline characteristics after Propensity Score Matching

	**Baseline characteristics after Propensity Score Matching**

Variables		LHR (n=63)	OHR (n=63)	p-value
**Age (years)**		70 (IQR 62-75)	67 (IQR 57-75)	0.5695(W)
**Sex**	Male	30 (47.62%)	28 (44.44%)	0.7207(C)
	Female	33 (52.38%)	35 (55.56%)	
**Height (cm)**		158 (IQR 150.1-165.0)	156 (IQR 149.7-165.0)	0.8224(W)
**Weight (kg)**		58.73 ± 12.54	58.88 ± 10.83	0.9442(T)
**Body mass index (kg/m^2)**		23.3 (IQR 20.9-25.8)	23.4 (IQR 21.6-24.7)	0.9455(W)
**Smoking**	No	57 (90.48%)	58 (92.06%)	0.5784(F)
	Past	2 (3.17%)	0 (0.00%)	
	Present	4 (6.35%)	5 (7.94%)	
**ASA classification**	1	7 (11.11%)	7 (11.11%)	0.9688(C)
	2	47 (74.60%)	46 (73.02%)	
	3	9 (14.29%)	10 (15.87%)	
**Diabetes**	Yes	10 (15.87%)	10 (15.87%)	1.0000(C)
	No	53 (84.13%)	53 (84.13%)	
**Hypertension**	Yes	39 (61.90%)	41 (65.08%)	0.7113(C)
	No	24 (38.10%)	22 (34.92%)	
**Heart disease**	Yes	12 (19.05%)	9 (14.29%)	0.4733(C)
	No	51 (80.95%)	54 (85.71%)	
**Pulmonary disease**	Yes	6 (9.52%)	4 (6.35%)	0.5098(C)
	No	57 (90.48%)	59 (93.65%)	
**Liver disease**	Yes	1 (1.59%)	1 (1.59%)	1.0000(F)
	No	62 (98.41%)	62 (98.41%)	
**Cerebrovascular disease**	Yes	7 (11.11%)	2 (3.17%)	0.1638(F)
	No	56 (88.89%)	61 (96.83%)	
**Cancer or benign**	Cancer	22 (34.92%)	18 (28.57%)	0.4440(C)
	benign	41 (65.08%)	45 (71.43%)	
**Hartmann method**	Open	7 (11.11%)	60 (95.24%)	<0.0001*(F)
	Laparoscopy	53 (84.13%)	1 (1.59%)	
	Conversion	3 (4.76%)	2 (3.17%)	

LHR, Laparoscopic Hartmann Reversal; OHR, Open Hartmann Reversal; ASA, American Society of Anesthesiologists; IQR, interquartile range

p-value: Independent t-test (T) or Wilcoxon rank sum test (W)

p-value: Chi-square test (C) or Fishers exact test (F)

Operation Related Factors

There were no differences in terms of stump length (p=0.925), adhesiolysis (p=0.503), anastomotic method (p=0.803), diversion (p=0.492), or surgery time (p=0.718) between the two groups (Table 3).

Table 3. Operation related factors

Variables		Operation related factors		
		**LHR (n=63)**	**OHR (n=63)**	**p-value**
**Stump length (cm)**		15 (IQR 10-20)	15 (IQR 10-20)	0.9254(W)
**Stump resection**	Yes	11 (17.46%)	12 (19.35%)	0.7846(C)
	No	52 (82.54%)	50 (80.65%)	
**Adhesiolysis**	Yes	49 (77.78%)	52 (82.54%)	0.5028(C)
	No	14 (22.22%)	11 (17.46%)	
**Combined resection**	Yes	9 (14.29%)	10 (15.87%)	0.8034(C)
	No	54 (85.71%)	53 (84.13%)	
**Distance from anal verge to anastomosis (cm)**		11 (IQR 8-15)	15 (IQR 9-17)	0.4869(W)
**Anastomotic method**	end-to-end	54 (85.71%)	53 (84.13%)	0.8034(C)
	Other	9 (14.29%)	10 (15.87%)	
**Circular stapler size (mm)**		28 (IQR 25-28)	28 (IQR 25-28)	0.1771(W)
**Diversion**	Yes	3 (4.76%)	6 (9.52%)	0.4915(F)
	No	60 (95.24%)	57 (90.48%)	
**Surgery time (min)**		210 (IQR 159-290)	233 (IQR 160-280)	0.7180(W)

LHR, Laparoscopic Hartmann Reversal; OHR, Open Hartmann Reversal; IQR, interquartile range

p-value: Independent t-test (T) or Wilcoxon rank sum test (W)

p-value: Chi-square test (C) or Fishers exact test (F)

Primary Outcome

POI frequencies with prevalence in the LHR and OHR groups were 3 (4.76%) and 14 (22.2%), respectively, with significant differences (p=0.004) (Table 4).

Table 4. Postoperative outcomes

		Postoperative outcomes		
**Variables**		**LHR (n=63)**	**OHR (n=63)**	**p-value**
**Length of stay (days)**		9 (IQR 8-11)	12 (IQR 10-16)	**<0.0001*(W)**
**Time to solid diet (days)**		4 (IQR 3-5)	6 (IQR 5-8)	**<0.0001*(W)**
**Clavien-Dindo classification**	<IIIa	60 (95.24%)	58 (92.06%)	0.7175(F)
	≧IIIa	3 (4.76%)	5 (7.94%)	
**Postoperative ileus**	Yes	3 (4.76%)	14 (22.22%)	**0.0041*(C)**
	No	60 (95.24%)	49 (77.78%)	
**Wound infection**	Yes	5 (7.94%)	9 (14.29%)	0.2568(C)
	No	58 (92.06%)	54 (85.71%)	
**Anastomotic stricture**	Yes	0 (0.00%)	2 (7.69%)	0.2189(F)
	No	29 (100.00%)	24 (92.31%)	
**Anastomotic leakage**	Yes	0 (0.00%)	0 (0.00%)	NA
	No	63 (100.00%)	63 (100.00%)	
**Intraabdominal abscess**	Yes	0 (0.00%)	0 (0.00%)	NA
	No	63 (100.00%)	63 (100.00%)	
**Incisional hernia**	Yes	11 (28.95%)	6 (15.38%)	0.1514(C)
	No	27 (71.05%)	33 (84.62%)	

LHR, Laparoscopic Hartmann Reversal; OHR, Open Hartmann Reversal

p-value: Independent t-test (T) or Wilcoxon rank sum test (W)

p-value: Chi-square test (C) or Fishers exact test (F)

In the LHR group, two POI patients showed improvement in their symptoms at 2 and 3 days, respectively, after L-tube insertion with parenteral nutritional support. However, one patient underwent laparoscopic adhesiolysis with transverse colectomy to resolve adhesions.

Of 13 POI patients in the OHR group, three patients showed spontaneous improvement in their symptoms without L-tube insertion. However, one patient underwent open adhesiolysis with T-colostomy to resolve the adhesion.

Secondary Outcomes

The median lengths of stay in the LHR and OHR groups were 9 and 12 days, respectively, showing a significantly shorter length of stay in the LHR group ($p<0.001$). In terms of the median time to solid diet, the LHR group showed a significantly shorter period than the OHR group (4 vs 6 days, $p<0.001$). There was no difference in postoperative complications between the two groups. During the median follow-up period of 346 days, there was no difference in the frequency of incisional hernia between the two groups ($p=0.151$) (Table 4).

Subgroup analysis

Of 246 patients who underwent open or conversion Hartmann's operation among the included 322 patients (Fig 1), 223 had undergone OHR and 23 had undergone LHR; the frequencies with the prevalence of POI between the two groups were 1 (4.4%) for LHR and 48 (21.5%) for OHR ($p=0.055$). In terms of the median time to solid diet, the LHR group (5 days) showed a significantly shorter time than the OHR group (6 days) ($p<0.001$). The median lengths of stay in the LHR and OHR groups were 9 and 12 days, respectively, showing a significantly shorter length of stay in the LHR group ($p<0.001$). Complications did not differ between the two groups (Supplement Table).

Discussion

Overall, the results of this study showed that LHR reduced POI frequency and decreased the time to solid diet and hospital stay compared to OHR. Similar results were observed in a group of patients who had previously undergone open Hartmann's operation in the subgroup analysis.

In colorectal surgery, the advantages of minimally invasive surgery, such as laparoscopic surgery, compared to open surgery include a more rapid recovery and fewer postoperative

complications, such as wound infection. As such, minimally invasive surgery is strongly recommended by the 2018 ERAS Society [17]. However, LHR is more difficult than general laparoscopic surgery because of the presence of intra-abdominal adhesions caused by a past surgical history [6-10]. Nevertheless, the current literature on laparoscopic Hartmann's reversal highlights the advantages of the laparoscopic approach, such as reducing major complications after surgery and lowering the anastomotic leakage rate [18].

POI is a complication that not only causes patient dissatisfaction, but also prolongs hospital stay [19]. Furthermore, randomized controlled trials using scintigraphy and radiological transit studies to investigate the effect of laparoscopic colorectal surgery on gastrointestinal function confirmed that gastrointestinal function was improved following this surgery [20-22]. LHR has also been reported to significantly reduce POI [13, 14]. Although these studies have the disadvantage of using single-center data, we once again confirmed that LHR plays an important role in reducing POI through propensity score matching using multi-center data.

In our study, LHR shortened both the time to soft diet initiation and the length of hospital stay. This finding agrees with that of a recent meta-analysis [18]. In addition, the recently revised guidelines from the American Society of Colon and Rectal Surgeons and the Society of American Gastrointestinal and Endoscopic Surgeons strongly recommend adopting a minimally invasive surgical approach because of its advantages in terms of bowel function and length of hospital stay during colorectal surgery [23]. Although LHR is more difficult to perform than general colorectal surgery, if surgeons with the requisite expertise are available, LHR should be performed as it is thought to be helpful in the early recovery of patients, such as speeding up time to solid diet, reducing postoperative pain, and shortening the length of hospital stay.

LHR is a challenging operation because of adhesions due to previous surgical history [6-10].

Accordingly, there are inevitable cases in which it is difficult to complete surgery with the laparoscopic approach. According to a meta-analysis, the average conversion rate is generally around 16.1%, but has been reported to reach up to 50% [24]. In our study, of a total of 104 patients who underwent the laparoscopic approach, 15 (14.4%) required conversion, showing results similar to those in the previous literature. Furthermore, on analyzing the difference between LHR, OHR, and conversion (Supplement Table), the conversion group had a longer hospitalization period (p=0.016) but was not affected by POI (p=0.1530) or time to solid diet (p>0.999). There were no differences in other complications among groups. The length of stay in the conversion group did not differ from that in the OHR group (p>0.999), and there was no difference in operation time (p=0.640) or other complications compared to that in the OHR group. This suggests that open conversion while using the laparoscopic approach does not affect the postoperative prognosis.

A recent meta-analysis reported that the frequency of incisional hernia was significantly lower in LHR than that in OHR (OR = 0.42, 95% CI [0.23–0.74], $p < 0.003$) [18]. However, in our study, we observed no difference in the frequency of incisional hernias between the two groups. This may be because only 38 of 63 patients (60.3%) in the LHR group and 39 of 63 patients (61.9%) in the OHR group underwent CT during the follow-up period. It seems necessary to confirm the difference in the frequency of incisional hernia through a multicenter study, including a long-term follow-up.

The primary strength of our study is that it was a multicenter propensity score-matched study. Although this was a retrospective study, post hoc power analysis using the observed differences in the primary outcome variable showed that the result was significant; moreover, the estimated power was confirmed to be approximately 82.6%. However, the present study had some limitations, including the inevitable inclusion of bias owing to the retrospective design. Second,

there was a lack of description in the records regarding the reason for patients undergoing Hartmann’s operation and the degree of adhesion. It is thought that specific reasons such as fecal contamination index and surgeon proficiency for Hartmann's operation may be related to the degree of adhesion, affecting the decision of the method of Hartmann reversal. Additionally, it is possible that selective bias occurred as laparoscopic Hartmann's operation was performed more often in the case of LHR. However, even in the subgroup analysis of the patient group who underwent Open Hartmann's operation, the advantages of LHR were similar to the study results of the entire patient group; therefore, our results support the current recommendations to proceed with the laparoscopic approach if possible.

Conclusion

In conclusion, this study showed that LHR can be performed safely, and has the advantages of reducing POI and length of hospital stay compared with OHR. Because of the limitations of this retrospective study, a multicenter randomized study with long-term follow-up is needed to verify our results.

Acknowledgements

It was supported by Uijeongbu St. Mary's Hospital Clinical Research Coordinating Center as part of the clinical trial activation project.

References

1. Bonjer HJ, Haglind E, Jeekel I, Kazemier G, Pahlman L, Hop WCJ et al. Laparoscopic surgery versus open surgery for colon cancer: short-term outcomes of a randomised trial. Lancet Oncology. 2005;6(7):477-84. doi:10.1016/S1470-2045(05)70221-7.

2. Guillou PJ, Quirke P, Thorpe H, Walker J, Jayne DG, Smith AM et al. Short-term endpoints

of conventional versus laparoscopic-assisted surgery in patients with colorectal cancer (MRC CLASICC trial): multicentre, randomised controlled trial. Lancet. 2005;365(9472):1718-26. doi:10.1016/S0140-6736(05)66545-2.

3. Leung KL, Kwok SP, Lam SC, Lee JF, Yiu RY, Ng SS et al. Laparoscopic resection of rectosigmoid carcinoma: prospective randomised trial. Lancet. 2004;363(9416):1187-92. doi:10.1016/S0140-6736(04)15947-3.

4. Sallinen V, Di Saverio S, Haukijarvi E, Juusela R, Wikstrom H, Koivukangas V et al. Laparoscopic versus open adhesiolysis for adhesive small bowel obstruction (LASSO): an international, multicentre, randomised, open-label trial. Lancet Gastroenterol Hepatol. 2019;4(4):278-86. doi:10.1016/S2468-1253(19)30016-0.

5. Gachabayov M, Oberkofler CE, Tuech JJ, Hahnloser D, Bergamaschi R. Resection with primary anastomosis vs nonrestorative resection for perforated diverticulitis with peritonitis: a systematic review and meta-analysis. Colorectal Dis. 2018;20(9):753-70. doi:10.1111/codi.14237.

6. Banerjee S, Leather AJM, Rennie JA, Samano N, Gonzalez JG, Papagrigoriadis S. Feasibility and morbidity of reversal of Hartmann's. Colorectal Disease. 2005;7(5):454-9. doi:10.1111/j.1463-1318.2005.00862.x.

7. Cellini C, Deeb AP, Sharma A, Monson JRT, Fleming FJ. Association between operative approach and complications in patients undergoing Hartmann's reversal. British Journal of Surgery. 2013;100(8):1094-9. doi:10.1002/bjs.9153.

8. Garber A, Hyman N, Osler T. Complications of Hartmann takedown in a decade of preferred primary anastomosis. American Journal of Surgery. 2014;207(1):60-4. doi:10.1016/j.amjsurg.2013.05.006.

9. Hess GF, Schafer J, Rosenthal R, Kettelhack C, Oertli D. Reversal after Hartmann's procedure in patients with complicated sigmoid diverticulitis. Colorectal Disease.

2017;19(6):582-8. doi:10.1111/codi.13553.
10. Hodgson R, An V, Stupart DA, Guest GD, Watters DAK. Who gets Hartmann's reversed in a regional centre? Surgeon-Journal of the Royal Colleges of Surgeons of Edinburgh and Ireland. 2016;14(4):184-9. doi:10.1016/j.surge.2014.11.001.
11. Clermonts SH, de Ruijter WM, van Loon YT, Wasowicz DK, Heisterkamp J, Maring JK et al. Reversal of Hartmann's procedure utilizing single-port laparoscopy: an attractive alternative to laparotomy. Surg Endosc. 2016;30(5):1894-901. doi:10.1007/s00464-015-4407-3.
12. Thambi P, Borowski DW, Sathasivam R, Obuobi RB, Viswanath YKS, Gill TS. Single-incision laparoscopic reversal of Hartmann's operation through the stoma site: comparative outcomes with conventional laparoscopic and open surgery. Colorectal Disease. 2019;21(7):833-40. doi:10.1111/codi.14617.
13. Ng DC, Guarino S, Yau SL, Fok BK, Cheung HY, Li MK et al. Laparoscopic reversal of Hartmann's procedure: safety and feasibility. Gastroenterol Rep (Oxf). 2013;1(2):149-52. doi:10.1093/gastro/got018.
14. Yang PF, Morgan MJ. Laparoscopic versus open reversal of Hartmann's procedure: a retrospective review. ANZ J Surg. 2014;84(12):965-9. doi:10.1111/ans.12667.
15. Delaney CP, Senagore AJ, Gerkin TM, Beard TL, Zingaro WM, Tomaszewski KJ et al. Association of surgical care practices with length of stay and use of clinical protocols after elective bowel resection: results of a national survey. Am J Surg. 2010;199(3):299-304. doi:10.1016/j.amjsurg.2009.08.027.
16. Kee HH, Sik JC, Hee LD, Cheol KH, Sik YC, Kyu PS et al. Anastomotic Stricture after Colorectal Stapled Anastomosis. J Korean Soc Coloproctol. 2000;16(3):198-203.
17. Gustafsson UO, Scott MJ, Hubner M, Nygren J, Demartines N, Francis N et al. Guidelines for Perioperative Care in Elective Colorectal Surgery: Enhanced Recovery After Surgery (ERAS((R))) Society Recommendations: 2018. World J Surg. 2019;43(3):659-95.

doi:10.1007/s00268-018-4844-y.

18. Chavrier D, Alves A, Menahem B. Is laparoscopy a reliable alternative to laparotomy in Hartmann's reversal? An updated meta-analysis. Techniques in Coloproctology. 2022;26(4):239-52. doi:10.1007/s10151-021-02560-2.

19. Stein SL. Perioperative management. Clin Colon Rectal Surg. 2013;26(3):137-8. doi:10.1055/s-0033-1351141.

20. Basse L, Madsen JL, Billesbolle P, Bardram L, Kehlet H. Gastrointestinal transit after laparoscopic versus open colonic resection. Surg Endosc. 2003;17(12):1919-22. doi:10.1007/s00464-003-9013-0.

21. Schwenk W, Bohm B, Haase O, Junghans T, Muller JM. Laparoscopic versus conventional colorectal resection: a prospective randomised study of postoperative ileus and early postoperative feeding. Langenbecks Arch Surg. 1998;383(1):49-55. doi:10.1007/s004230050091.

22. van Bree SH, Vlug MS, Bemelman WA, Hollmann MW, Ubbink DT, Zwinderman AH et al. Faster recovery of gastrointestinal transit after laparoscopy and fast-track care in patients undergoing colonic surgery. Gastroenterology. 2011;141(3):872-80 e1-4. doi:10.1053/j.gastro.2011.05.034.

23. Irani JL, Hedrick TL, Miller TE, Lee LWC, Steinhagen E, Shogan BD et al. Clinical practice guidelines for enhanced recovery after colon and rectal surgery from the American Society of Colon and Rectal Surgeons and the Society of American Gastrointestinal and Endoscopic Surgeons. Surg Endosc. 2022. doi:10.1007/s00464-022-09758-x.

24. Celentano V, Giglio MC, Bucci L. Laparoscopic versus open Hartmann's reversal: a systematic review and meta-analysis. Int J Colorectal Dis. 2015;30(12):1603-15. doi:10.1007/s00384-015-2325-4.

Supporting information

S1 Table. Baseline characteristics

S2 Table. Operation related factors

S3 Table. Postoperative outcomes

S4 Table. Baseline characteristics between LHR, conversion and OHR

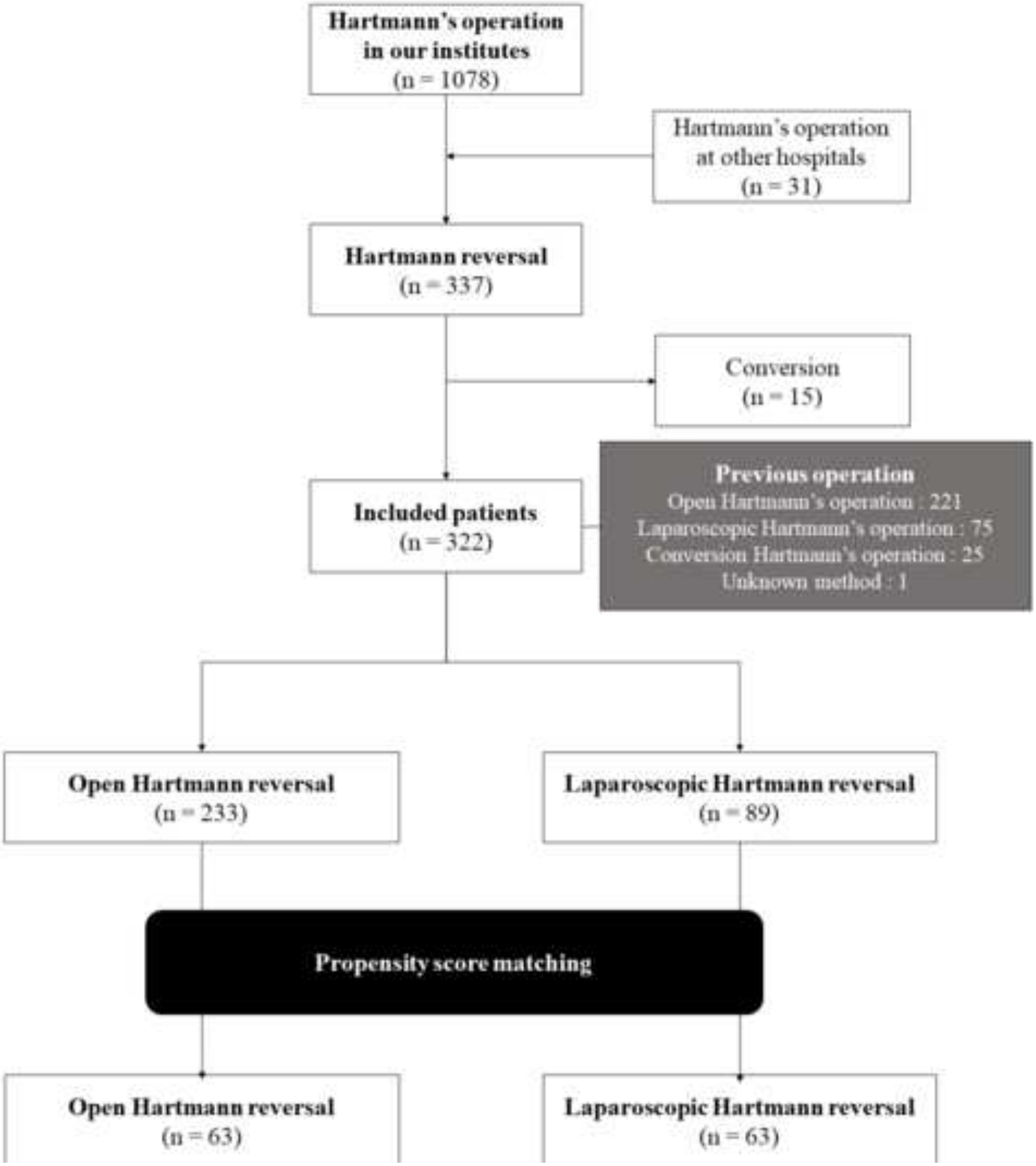
Hartmann's operation in our institutes (n = 1078)
Hartmann's operation at other hospitals (n = 31)
Hartmann reversal (n = 337)
Conversion (n = 15)
Included patients (n = 322)
Previous operation
Open Hartmann's operation : 221
Laparoscopic Hartmann's operation : 75
Conversion Hartmann's operation : 25
Unknown method : 1
Open Hartmann reversal (n = 233)
Laparoscopic Hartmann reversal (n = 89)
Propensity score matching
Open Hartmann reversal (n = 63)
Laparoscopic Hartmann reversal (n = 63)

Click here to access/download
Supporting Information
Supplement Tables.xlsx

Postoperative effects of laparoscopic Hartmann reversal: A multicenter propensity score matched study.

Kil-yong Lee[1], Jaeim Lee[1*], Seong Taek Oh[1], Chul Seung Lee[2], Nam Suk Kim[3], Ju Myung Song[4], Ri-Na Yoo[5], Byung Jo Choi[6]

[1]Department of Surgery, Uijeongbu St. Mary’s Hospital, College of Medicine, The Catholic University of Korea

[2]Department of Surgery, Seoul St. Mary's Hospital, College of Medicine, The Catholic University of Korea

[3]Department of Surgery, Yeouido St. Mary's Hospital, College of Medicine, The Catholic University of Korea

[4]Department of Surgery, Incheon St. Mary’s Hospital, College of Medicine, The Catholic University of Korea

[5]Department of Surgery, St. Vincent’s Hospital, College of Medicine, The Catholic University of Korea

[6]Department of Surgery, Daejeon St. Mary's Hospital, College of Medicine, The Catholic University of Korea

* Corresponding author:

E-mail: lji96@catholic.ac.kr (JIL)

Abstract

Background: Although the advantages of laparoscopic Hartmann reversal (LHR) compared to open Hartmann reversal (OHR) have been reported in the literature, the number of multicenter studies with good matching investigating this topic is rare. In the present study, we aimed to confirm the advantages of LHR in terms of short-term outcomes through propensity score matching of LHR and OHR groups, using data collected from multiple institutions.

Methods: Patients who underwent Hartmann reversal at six institutions under the Catholic Medical Center of the Catholic University of Korea between January 1, 2005, and December 31, 2021, were included. The patients were divided into the LHR and OHR groups based on the technique used. The two groups were matched using propensity score matching (1:1 ratio, logistic regression with the nearest-neighbor method). The primary outcome was postoperative ileus (POI) frequency, and secondary outcomes were time to solid diet (days) and length of stay (days).

Results: Among 337 patients, propensity score matching was performed on 322, after excluding 15 who had undergone open conversion**.** Of these, 63 patients were assigned to each group through propensity score matching. There was no difference in the frequency of adhesiolysis (77.8% vs. 82.5%, p=0.503) or the operation time. (210 (IQR 159-290) vs. 233 (IQR 160-280), p=0.718) between the two groups. As the primary outcome, the LHR group showed significantly lower POI frequency than the OHR group. (4.8% vs. 22.2%, p=0.0041) Regarding the secondary outcomes, the LHR group showed a shorter period to solid diet than the OHR group. The length of hospital stay was also significantly shorter in the LHR group (4 vs. 6, p < 0.0001; 9 vs. 12, p<0.0001).

Conclusion: LHR is an effective method to ensure faster recovery of patients after surgery compared to OHR.

Keywords: colon, colostomy, colorectal surgery, laparoscopy

Introduction

Laparoscopic colorectal surgery for colorectal disease has several advantages, including a reduction in both postoperative pain and hospital stay [1-3]. Furthermore, even highly difficult laparoscopic surgeries such as those of adhesive small bowel obstruction can be safely performed [4]. Hartmann's operation is performed when primary anastomosis of the colon is difficult due to severe peritonitis caused by left colonic perforation or unstable vital signs [5], and Hartmann reversal is often performed in some patients who have undergone Hartmann’s procedure. However, there are cases in which surgeons hesitate to use laparoscopic surgery because of the difficulty of detachment due to peritoneal adhesions caused by a history of previous surgery [6-10]. However, it has been reported that laparoscopic surgery can be performed safely even with single incision during Hartmann reversal [11, 5, 12]. Nevertheless, there is debate regarding the merits of laparoscopic Hartmann’s reversal over open procedures due to the lack of randomized controlled studies.

To address this knowledge gap, in the present study, we aimed to confirm the advantages of laparoscopic Hartmann reversal (LHR) by confirming the difference in postoperative complications using propensity score matching in two groups of patients (laparoscopic vs open approaches) among patients undergoing Hartmann reversal at several centers.

Methods

This is a multicenter retrospective cohort study. This study was approved by the institutional review board (IRB) of the Catholic University of Korea and was performed in accordance with the IRB's guidelines and regulations. The requirement for informed consent was waived by the IRB.

Patients

Patients who underwent Hartman's reversal at six institutions under the Catholic Medical Center of the Catholic University of Korea between January 1, 2005 and December 31, 2021, were included. The exclusion criteria were conversion and missing data for the covariates used in propensity score matching. All the data were retrospectively reviewed.

As reported by Ng et al. [13] and Yang et al. [14], when the sample size was calculated with 80% power and alpha 0.05, the minimum sample size required for each group was 60 patients.

Definition

Postoperative ileus (POI) was defined as any situation that requires a return to "nil per os" or the insertion of a nasogastric tube (NG) [15]. Stump length was measured using sigmoidoscopy prior to surgery. Regarding incisional hernia, defined as a hernia comprising a protruding mass observed from the surgical scar during the follow-up period and confirmed by CT, only patients who underwent CT during the follow-up period to check for incisional hernia were included. Anastomotic stricture was defined as the inability to pass a 13.2-mm colonoscope through the colon, or the feeling of resistance during its passage [16].

Outcomes

The primary outcome was POI frequency, and the secondary outcomes were time to solid diet (days), length of hospital stay (days), and postoperative complications.

Statistical analysis

Patients were divided into two groups: the LHR and the open Hartmann reversal (OHR) groups for comparison of the clinical characteristics. Continuous variables were assessed using the independent t-test or Wilcoxon rank sum test, and discontinuous variables were analyzed using the Chi-square test and Fisher exact test.

Propensity score matching (1:1 ratio using logistic regression with the nearest-neighbor method) was applied to correct for factors that differed between the two groups. The covariates were age, sex, body mass index, smoking, diabetes, hypertension, heart disease, pulmonary disease, liver disease, cerebrovascular disease, stump length, cause of perforation (cancer or benign), combined resection, anastomosis method, and stapler size.

Statistical analyses were performed using SAS ver 9.4 (SAS Institute Inc., Cary, NC, USA). Statistical significance was set at $p<0.05$.

Results

Of the 337 patients who underwent Hartmann's reversal, 322 were included in this study, after excluding 15 who underwent conversion. Among the 322 included patients, 89 underwent LHR and 233 underwent OHR (Fig 1). The baseline characteristics are shown in Table 1. After propensity score matching, 63 participants were assigned to each group; their baseline characteristics are shown in Table 2. The post-matching patient number of this study was shown to be 82% in power analysis, which was considered an appropriate sample size.

Fig. 1 Flow diagram of patient selection

Table 1. Baseline characteristics before Propensity Score Matching

	Baseline characteristics before Propensity Score Matching

Variables		LHR (n=89)	OHR (n=233)	p-value
**Age (years)**		69 (IQR 59-75)	66.5 (IQR 57-74)	0.2300(W)
**Sex**	Male	45 (50.56%)	112 (48.07%)	0.6890(C)
	Female	44 (49.44%)	121 (51.93%)	
**Height (cm)**		160 (IQR 151.2-165.2)	157.6 (IQR 151.7-165.0)	0.7861(W)
**Weight (kg)**		58 (IQR 50.6-65.3)	58.7 (IQR 52.0-65.6)	0.8561(W)
**Body mass index (kg/m^2)**		23.2 (IQR 20.9-25.8)	23.4 (IQR 21.2-25.5)	0.8027(W)
**Smoking**	No	76 (85.39%)	192 (82.40%)	0.6819(C)
	Past	5 (5.62%)	12 (5.15%)	
	Present	8 (8.99%)	29 (12.45%)	
**ASA classification**	1	9 (10.23%)	33 (14.16%)	0.6119(C)
	2	68 (77.27%)	169 (72.53%)	
	3	11 (12.50%)	31 (13.30%)	
**Diabetes**	Yes	16 (17.98%)	45 (19.31%)	0.7844(C)
	No	73 (82.02%)	188 (80.69%)	
**Hypertension**	Yes	54 (60.67%)	127 (54.51%)	0.3185(C)
	No	35 (39.33%)	106 (45.49%)	
**Heart disease**	Yes	16 (17.98%)	31 (13.30%)	0.2882(C)
	No	73 (82.02%)	202 (86.70%)	
**Pulmonary disease**	Yes	12 (13.48%)	20 (8.58%)	0.1888(C)
	No	77 (86.52%)	213 (91.42%)	
**Liver disease**	Yes	3 (3.37%)	6 (2.58%)	0.7113(F)
	No	86 (96.63%)	227 (97.42%)	
**Cerebrovascular disease**	Yes	10 (11.24%)	11 (4.72%)	0.0342*(C)
	No	79 (88.76%)	222 (95.28%)	
**Cancer or benign**	Cancer	31 (34.83%)	80 (34.33%)	0.9332(C)
	benign	58 (65.17%)	153 (65.67%)	
**Hartmann method**	Open	19 (21.35%)	202 (87.07%)	<0.0001*(C)
	Laparoscopy	66 (74.16%)	9 (3.88%)	
	Conversion	4 (4.49%)	21 (9.05%)	

LHR, Laparoscopic Hartmann Reversal; OHR, Open Hartmann Reversal; ASA, American Society of Anesthesiologists; IQR, interquartile range

p-value: Independent t-test (T) or Wilcoxon rank sum test (W)

p-value: Chi-square test (C) or Fishers exact test (F)

Table 2. Baseline characteristics after Propensity Score Matching

	Baseline characteristics after Propensity Score Matching

**Variables**		**LHR (n=63)**	**OHR (n=63)**	**p-value**
**Age (years)**		70 (IQR 62-75)	67 (IQR 57-75)	0.5695(W)
**Sex**	Male	30 (47.62%)	28 (44.44%)	0.7207(C)
	Female	33 (52.38%)	35 (55.56%)	
**Height (cm)**		158 (IQR 150.1-165.0)	156 (IQR 149.7-165.0)	0.8224(W)
**Weight (kg)**		58.73 ± 12.54	58.88 ± 10.83	0.9442(T)
**Body mass index (kg/m^2)**		23.3 (IQR 20.9-25.8)	23.4 (IQR 21.6-24.7)	0.9455(W)
**Smoking**	No	57 (90.48%)	58 (92.06%)	0.5784(F)
	Past	2 (3.17%)	0 (0.00%)	
	Present	4 (6.35%)	5 (7.94%)	
**ASA classification**	1	7 (11.11%)	7 (11.11%)	0.9688(C)
	2	47 (74.60%)	46 (73.02%)	
	3	9 (14.29%)	10 (15.87%)	
**Diabetes**	Yes	10 (15.87%)	10 (15.87%)	1.0000(C)
	No	53 (84.13%)	53 (84.13%)	
**Hypertension**	Yes	39 (61.90%)	41 (65.08%)	0.7113(C)
	No	24 (38.10%)	22 (34.92%)	
**Heart disease**	Yes	12 (19.05%)	9 (14.29%)	0.4733(C)
	No	51 (80.95%)	54 (85.71%)	
**Pulmonary disease**	Yes	6 (9.52%)	4 (6.35%)	0.5098(C)
	No	57 (90.48%)	59 (93.65%)	
**Liver disease**	Yes	1 (1.59%)	1 (1.59%)	1.0000(F)
	No	62 (98.41%)	62 (98.41%)	
**Cerebrovascular disease**	Yes	7 (11.11%)	2 (3.17%)	0.1638(F)
	No	56 (88.89%)	61 (96.83%)	
**Cancer or benign**	Cancer	22 (34.92%)	18 (28.57%)	0.4440(C)
	benign	41 (65.08%)	45 (71.43%)	
**Hartmann method**	Open	7 (11.11%)	60 (95.24%)	<0.0001*(F)
	Laparoscopy	53 (84.13%)	1 (1.59%)	
	Conversion	3 (4.76%)	2 (3.17%)	

LHR, Laparoscopic Hartmann Reversal; OHR, Open Hartmann Reversal; ASA, American Society of Anesthesiologists; IQR, interquartile range

p-value: Independent t-test (T) or Wilcoxon rank sum test (W)

p-value: Chi-square test (C) or Fishers exact test (F)

Operation Related Factors

There were no differences in terms of stump length (p=0.925), adhesiolysis (p=0.503), anastomotic method (p=0.803), diversion (p=0.492), or surgery time (p=0.718) between the two groups (Table 3).

Table 3. Operation related factors

		Operation related factors		
**Variables**		**LHR (n=63)**	**OHR (n=63)**	**p-value**
**Stump length (cm)**		15 (IQR 10-20)	15 (IQR 10-20)	0.9254(W)
**Stump resection**	Yes	11 (17.46%)	12 (19.35%)	0.7846(C)
	No	52 (82.54%)	50 (80.65%)	
**Adhesiolysis**	Yes	49 (77.78%)	52 (82.54%)	0.5028(C)
	No	14 (22.22%)	11 (17.46%)	
**Combined resection**	Yes	9 (14.29%)	10 (15.87%)	0.8034(C)
	No	54 (85.71%)	53 (84.13%)	
**Distance from anal verge to anastomosis (cm)**		11 (IQR 8-15)	15 (IQR 9-17)	0.4869(W)
**Anastomotic method**	end-to-end	54 (85.71%)	53 (84.13%)	0.8034(C)
	Other	9 (14.29%)	10 (15.87%)	
**Circular stapler size (mm)**		28 (IQR 25-28)	28 (IQR 25-28)	0.1771(W)
**Diversion**	Yes	3 (4.76%)	6 (9.52%)	0.4915(F)
	No	60 (95.24%)	57 (90.48%)	
**Surgery time (min)**		210 (IQR 159-290)	233 (IQR 160-280)	0.7180(W)

LHR, Laparoscopic Hartmann Reversal; OHR, Open Hartmann Reversal; IQR, interquartile range

p-value: Independent t-test (T) or Wilcoxon rank sum test (W)

p-value: Chi-square test (C) or Fishers exact test (F)

Primary Outcome

POI frequencies with prevalence in the LHR and OHR groups were 3 (4.76%) and 14 (22.2%), respectively, with significant differences (p=0.004) (Table 4).

Table 4. Postoperative outcomes

		Postoperative outcomes		
**Variables**		**LHR (n=63)**	**OHR (n=63)**	**p-value**
**Length of stay (days)**		9 (IQR 8-11)	12 (IQR 10-16)	**<0.0001*(W)**
**Time to solid diet (days)**		4 (IQR 3-5)	6 (IQR 5-8)	**<0.0001*(W)**
**Clavien-Dindo classification**	<IIIa	60 (95.24%)	58 (92.06%)	0.7175(F)
	≧IIIa	3 (4.76%)	5 (7.94%)	
**Postoperative ileus**	Yes	3 (4.76%)	14 (22.22%)	**0.0041*(C)**
	No	60 (95.24%)	49 (77.78%)	
**Wound infection**	Yes	5 (7.94%)	9 (14.29%)	0.2568(C)
	No	58 (92.06%)	54 (85.71%)	
**Anastomotic stricture**	Yes	0 (0.00%)	2 (7.69%)	0.2189(F)
	No	29 (100.00%)	24 (92.31%)	
**Anastomotic leakage**	Yes	0 (0.00%)	0 (0.00%)	NA
	No	63 (100.00%)	63 (100.00%)	
**Intraabdominal abscess**	Yes	0 (0.00%)	0 (0.00%)	NA
	No	63 (100.00%)	63 (100.00%)	
**Incisional hernia**	Yes	11 (28.95%)	6 (15.38%)	0.1514(C)
	No	27 (71.05%)	33 (84.62%)	

LHR, Laparoscopic Hartmann Reversal; OHR, Open Hartmann Reversal

p-value: Independent t-test (T) or Wilcoxon rank sum test (W)

p-value: Chi-square test (C) or Fishers exact test (F)

In the LHR group, two POI patients showed improvement in their symptoms at 2 and 3 days, respectively, after L-tube insertion with parenteral nutritional support. However, one patient underwent laparoscopic adhesiolysis with transverse colectomy to resolve adhesions.

Of 13 POI patients in the OHR group, three patients showed spontaneous improvement in their symptoms without L-tube insertion. However, one patient underwent open adhesiolysis with T-colostomy to resolve the adhesion.

Secondary Outcomes

The median lengths of stay in the LHR and OHR groups were 9 and 12 days, respectively, showing a significantly shorter length of stay in the LHR group (p<0.001). In terms of the median time to solid diet, the LHR group showed a significantly shorter period than the OHR group (4 vs 6 days, p<0.001). There was no difference in postoperative complications between the two groups. During the median follow-up period of 346 days, there was no difference in the frequency of incisional hernia between the two groups (p=0.151) (Table 4).

Subgroup analysis

Of 246 patients who underwent open or conversion Hartmann’s operation among the included 322 patients (Fig 1), 223 had undergone OHR and 23 had undergone LHR; the frequencies with the prevalence of POI between the two groups were 1 (4.4%) for LHR and 48 (21.5%) for OHR (p=0.055). In terms of the median time to solid diet, the LHR group (5 days) showed a significantly shorter time than the OHR group (6 days) (p<0.001). The median lengths of stay in the LHR and OHR groups were 9 and 12 days, respectively, showing a significantly shorter length of stay in the LHR group (p<0.001). Complications did not differ between the two groups (Supplement Table).

Discussion

Overall, the results of this study showed that LHR reduced POI frequency and decreased the time to solid diet and hospital stay compared to OHR. Similar results were observed in a group of patients who had previously undergone open Hartmann's operation in the subgroup analysis.

In colorectal surgery, the advantages of minimally invasive surgery, such as laparoscopic surgery, compared to open surgery include a more rapid recovery and fewer postoperative

complications, such as wound infection. As such, minimally invasive surgery is strongly recommended by the 2018 ERAS Society [17]. However, LHR is more difficult than general laparoscopic surgery because of the presence of intra-abdominal adhesions caused by a past surgical history [6-10]. Nevertheless, the current literature on laparoscopic Hartmann's reversal highlights the advantages of the laparoscopic approach, such as reducing major complications after surgery and lowering the anastomotic leakage rate [18].

POI is a complication that not only causes patient dissatisfaction, but also prolongs hospital stay [19]. Furthermore, randomized controlled trials using scintigraphy and radiological transit studies to investigate the effect of laparoscopic colorectal surgery on gastrointestinal function confirmed that gastrointestinal function was improved following this surgery [20-22]. LHR has also been reported to significantly reduce POI [13, 14]. Although these studies have the disadvantage of using single-center data, we once again confirmed that LHR plays an important role in reducing POI through propensity score matching using multi-center data.

In our study, LHR shortened both the time to soft diet initiation and the length of hospital stay. This finding agrees with that of a recent meta-analysis [18]. In addition, the recently revised guidelines from the American Society of Colon and Rectal Surgeons and the Society of American Gastrointestinal and Endoscopic Surgeons strongly recommend adopting a minimally invasive surgical approach because of its advantages in terms of bowel function and length of hospital stay during colorectal surgery [23]. Although LHR is more difficult to perform than general colorectal surgery, if surgeons with the requisite expertise are available, LHR should be performed as it is thought to be helpful in the early recovery of patients, such as speeding up time to solid diet, reducing postoperative pain, and shortening the length of hospital stay.

LHR is a challenging operation because of adhesions due to previous surgical history [6-10].

Accordingly, there are inevitable cases in which it is difficult to complete surgery with the laparoscopic approach. According to a meta-analysis, the average conversion rate is generally around 16.1%, but has been reported to reach up to 50% [24]. In our study, of a total of 104 patients who underwent the laparoscopic approach, 15 (14.4%) required conversion, showing results similar to those in the previous literature. Furthermore, on analyzing the difference between LHR, OHR, and conversion (Supplement Table), the conversion group had a longer hospitalization period (p=0.016) but was not affected by POI (p=0.1530) or time to solid diet (p>0.999). There were no differences in other complications among groups. The length of stay in the conversion group did not differ from that in the OHR group (p>0.999), and there was no difference in operation time (p=0.640) or other complications compared to that in the OHR group. This suggests that open conversion while using the laparoscopic approach does not affect the postoperative prognosis.

A recent meta-analysis reported that the frequency of incisional hernia was significantly lower in LHR than that in OHR (OR = 0.42, 95% CI [0.23–0.74], p < 0.003) [18]. However, in our study, we observed no difference in the frequency of incisional hernias between the two groups. This may be because only 38 of 63 patients (60.3%) in the LHR group and 39 of 63 patients (61.9%) in the OHR group underwent CT during the follow-up period. It seems necessary to confirm the difference in the frequency of incisional hernia through a multicenter study, including a long-term follow-up.

The primary strength of our study is that it was a multicenter propensity score-matched study. Although this was a retrospective study, post hoc power analysis using the observed differences in the primary outcome variable showed that the result was significant; moreover, the estimated power was confirmed to be approximately 82.6%. However, the present study had some limitations, including the inevitable inclusion of bias owing to the retrospective design. Second,

there was a lack of description in the records regarding the reason for patients undergoing Hartmann's operation and the degree of adhesion. It is thought that specific reasons such as fecal contamination index and surgeon proficiency for Hartmann's operation may be related to the degree of adhesion, affecting the decision of the method of Hartmann reversal. Additionally, it is possible that selective bias occurred as laparoscopic Hartmann's operation was performed more often in the case of LHR. However, even in the subgroup analysis of the patient group who underwent Open Hartmann's operation, the advantages of LHR were similar to the study results of the entire patient group; therefore, our results support the current recommendations to proceed with the laparoscopic approach if possible.

Conclusion

In conclusion, this study showed that LHR can be performed safely, and has the advantages of reducing POI and length of hospital stay compared with OHR. Because of the limitations of this retrospective study, a multicenter randomized study with long-term follow-up is needed to verify our results.

Acknowledgements

It was supported by Uijeongbu St. Mary's Hospital Clinical Research Coordinating Center as part of the clinical trial activation project.

References

1. Bonjer HJ, Haglind E, Jeekel I, Kazemier G, Pahlman L, Hop WCJ et al. Laparoscopic surgery versus open surgery for colon cancer: short-term outcomes of a randomised trial. Lancet Oncology. 2005;6(7):477-84. doi:10.1016/S1470-2045(05)70221-7.

2. Guillou PJ, Quirke P, Thorpe H, Walker J, Jayne DG, Smith AM et al. Short-term endpoints

of conventional versus laparoscopic-assisted surgery in patients with colorectal cancer (MRC CLASICC trial): multicentre, randomised controlled trial. Lancet. 2005;365(9472):1718-26. doi:10.1016/S0140-6736(05)66545-2.

3. Leung KL, Kwok SP, Lam SC, Lee JF, Yiu RY, Ng SS et al. Laparoscopic resection of rectosigmoid carcinoma: prospective randomised trial. Lancet. 2004;363(9416):1187-92. doi:10.1016/S0140-6736(04)15947-3.

4. Sallinen V, Di Saverio S, Haukijarvi E, Juusela R, Wikstrom H, Koivukangas V et al. Laparoscopic versus open adhesiolysis for adhesive small bowel obstruction (LASSO): an international, multicentre, randomised, open-label trial. Lancet Gastroenterol Hepatol. 2019;4(4):278-86. doi:10.1016/S2468-1253(19)30016-0.

5. Gachabayov M, Oberkofler CE, Tuech JJ, Hahnloser D, Bergamaschi R. Resection with primary anastomosis vs nonrestorative resection for perforated diverticulitis with peritonitis: a systematic review and meta-analysis. Colorectal Dis. 2018;20(9):753-70. doi:10.1111/codi.14237.

6. Banerjee S, Leather AJM, Rennie JA, Samano N, Gonzalez JG, Papagrigoriadis S. Feasibility and morbidity of reversal of Hartmann's. Colorectal Disease. 2005;7(5):454-9. doi:10.1111/j.1463-1318.2005.00862.x.

7. Cellini C, Deeb AP, Sharma A, Monson JRT, Fleming FJ. Association between operative approach and complications in patients undergoing Hartmann's reversal. British Journal of Surgery. 2013;100(8):1094-9. doi:10.1002/bjs.9153.

8. Garber A, Hyman N, Osler T. Complications of Hartmann takedown in a decade of preferred primary anastomosis. American Journal of Surgery. 2014;207(1):60-4. doi:10.1016/j.amjsurg.2013.05.006.

9. Hess GF, Schafer J, Rosenthal R, Kettelhack C, Oertli D. Reversal after Hartmann's procedure in patients with complicated sigmoid diverticulitis. Colorectal Disease.

2017;19(6):582-8. doi:10.1111/codi.13553.
10. Hodgson R, An V, Stupart DA, Guest GD, Watters DAK. Who gets Hartmann's reversed in a regional centre? Surgeon-Journal of the Royal Colleges of Surgeons of Edinburgh and Ireland. 2016;14(4):184-9. doi:10.1016/j.surge.2014.11.001.
11. Clermonts SH, de Ruijter WM, van Loon YT, Wasowicz DK, Heisterkamp J, Maring JK et al. Reversal of Hartmann's procedure utilizing single-port laparoscopy: an attractive alternative to laparotomy. Surg Endosc. 2016;30(5):1894-901. doi:10.1007/s00464-015-4407-3.
12. Thambi P, Borowski DW, Sathasivam R, Obuobi RB, Viswanath YKS, Gill TS. Single-incision laparoscopic reversal of Hartmann's operation through the stoma site: comparative outcomes with conventional laparoscopic and open surgery. Colorectal Disease. 2019;21(7):833-40. doi:10.1111/codi.14617.
13. Ng DC, Guarino S, Yau SL, Fok BK, Cheung HY, Li MK et al. Laparoscopic reversal of Hartmann's procedure: safety and feasibility. Gastroenterol Rep (Oxf). 2013;1(2):149-52. doi:10.1093/gastro/got018.
14. Yang PF, Morgan MJ. Laparoscopic versus open reversal of Hartmann's procedure: a retrospective review. ANZ J Surg. 2014;84(12):965-9. doi:10.1111/ans.12667.
15. Delaney CP, Senagore AJ, Gerkin TM, Beard TL, Zingaro WM, Tomaszewski KJ et al. Association of surgical care practices with length of stay and use of clinical protocols after elective bowel resection: results of a national survey. Am J Surg. 2010;199(3):299-304. doi:10.1016/j.amjsurg.2009.08.027.
16. Kee HH, Sik JC, Hee LD, Cheol KH, Sik YC, Kyu PS et al. Anastomotic Stricture after Colorectal Stapled Anastomosis. J Korean Soc Coloproctol. 2000;16(3):198-203.
17. Gustafsson UO, Scott MJ, Hubner M, Nygren J, Demartines N, Francis N et al. Guidelines for Perioperative Care in Elective Colorectal Surgery: Enhanced Recovery After Surgery (ERAS((R))) Society Recommendations: 2018. World J Surg. 2019;43(3):659-95.

doi:10.1007/s00268-018-4844-y.

18. Chavrier D, Alves A, Menahem B. Is laparoscopy a reliable alternative to laparotomy in Hartmann's reversal? An updated meta-analysis. Techniques in Coloproctology. 2022;26(4):239-52. doi:10.1007/s10151-021-02560-2.

19. Stein SL. Perioperative management. Clin Colon Rectal Surg. 2013;26(3):137-8. doi:10.1055/s-0033-1351141.

20. Basse L, Madsen JL, Billesbolle P, Bardram L, Kehlet H. Gastrointestinal transit after laparoscopic versus open colonic resection. Surg Endosc. 2003;17(12):1919-22. doi:10.1007/s00464-003-9013-0.

21. Schwenk W, Bohm B, Haase O, Junghans T, Muller JM. Laparoscopic versus conventional colorectal resection: a prospective randomised study of postoperative ileus and early postoperative feeding. Langenbecks Arch Surg. 1998;383(1):49-55. doi:10.1007/s004230050091.

22. van Bree SH, Vlug MS, Bemelman WA, Hollmann MW, Ubbink DT, Zwinderman AH et al. Faster recovery of gastrointestinal transit after laparoscopy and fast-track care in patients undergoing colonic surgery. Gastroenterology. 2011;141(3):872-80 e1-4. doi:10.1053/j.gastro.2011.05.034.

23. Irani JL, Hedrick TL, Miller TE, Lee LWC, Steinhagen E, Shogan BD et al. Clinical practice guidelines for enhanced recovery after colon and rectal surgery from the American Society of Colon and Rectal Surgeons and the Society of American Gastrointestinal and Endoscopic Surgeons. Surg Endosc. 2022. doi:10.1007/s00464-022-09758-x.

24. Celentano V, Giglio MC, Bucci L. Laparoscopic versus open Hartmann's reversal: a systematic review and meta-analysis. Int J Colorectal Dis. 2015;30(12):1603-15. doi:10.1007/s00384-015-2325-4.

Supporting information

S1 Table. Baseline characteristics

S2 Table. Operation related factors

S3 Table. Postoperative outcomes

S4 Table. Baseline characteristics between LHR, conversion and OHR

Review Comments to the Author

Reviewer #1: Title: Postoperative effects of laparoscopic Hartmann reversal: A multicenter propensity

score matched study.

The title is in line with study objectives and the results

Abstract: Structured and well written

Background: Detailed information on statement of problem as well as rational for the study clearly presented.

Methods: Fairly well described.

• Comment: Study design should be mentioned even though it is apparent from the methodology.

- ➔ We thank the reviewer for the considerate comments. Accordingly, we have indicated the study design at the beginning of the methods.

• Comment: Minimum sample size for the study was not calculated. This is desirable, as this will show whether the sample size in this study meets the minimum calculated sample size. If the sample size used is less than the calculated minimum sample size, then this can be a limitation in the study.

- ➔ According to the reviewer's comments, we have calculated sample size. As reported by Ng et al. [1] and Yang et al. [2], with 80% power and alpha 0.05, the sample size required for each group was 60 patients. Therefore, in this study, the number of samples included is appropriate, with a total of 126 patients and 63 patients in each group; the power was confirmed to be about 82% in the power analysis. This information has been added it in the "Patients" and "Results" section.

References

1. Ng DC, Guarino S, Yau SL, Fok BK, Cheung HY, Li MK et al. Laparoscopic reversal of Hartmann's procedure: safety and feasibility. Gastroenterol Rep (Oxf). 2013;1(2):149-52. doi:10.1093/gastro/got018.

2. Yang PF, Morgan MJ. Laparoscopic versus open reversal of Hartmann's procedure: a retrospective review. ANZ J Surg. 2014;84(12):965-9. doi:10.1111/ans.12667.

Result: Well written in details with relevant tables and figures

• Comment: The first column for most of the tables are without a label

➔ Accordingly, we have added the label to the first column.

• **Comment: The state number of days for POI, hospital stay, etc are not really clear. Are these mean values or median? If it is mean value, please indicate it in the sentence and provide the standard deviation (SD) as well**.

➔ The number of days for hospital stay and the time to solid diet are described as the median. These have been added to the results section.

• **Comment: Line 126 - Frequency cannot be in percentage. Please correct it. Is it the prevalence?**

➔ This indicates the prevalence of POIs in each group. According to the reviewer's comment, we have changed it to the number of occurrences as well as the percentage.

Discussion: The study findings are well discussed, with study limitations provided.

Conclusion: Clearly written with appropriate recommendation.

Reviewer #2: The author focused to the short-term outcomes of reversal Hartmann operation and compared the advantages of laparoscopic and open surgery in this operation. They enrolled the patients from multi-institutions retrospectively and used the propensity score matched analysis for deep statistical analysis. From the analysis, the author have concluded that laparoscopic Hartmann reversal (LHR) is better method for fast recovery from the surgery and to prevent the postoprative bowel obstrution than open Hartmann reversal (OHR). Overall, the analysis seems to be fair and the results of this manuscript are similar to the previous studies and I agree with it. As the author pointed out, the study which reported the outcome of Hartmann reversal operation is not that many and these previous publications are mainly from single institution. This presented study is multi-instituional study with propensity score matced analysis and these background seems to strengthen the result compared to the previous studies. However, there are several concerns in this study which I want to point out.

1. **Comment: As the author pointed out, the peritoneal adhesion will be the challenge to perform LHR. I feel that the conversion rate of LHR is another important outcome and I do not understand why the author excluded the 15 conversion cases from the analysis. It is more important to know how the LHR is difficult and conversion to open surgery may happen. Looking into the result, it seems that about 15% of patients were converted to open surgery and I recommend the author to analyze and discuss more deeply about it.**

→ We appreciate the reviewer's considerate comments. The initial intention of the group that underwent conversion was LHR; thus, if it were analyzed by intention-to-treat (ITT) method, this group should be analyzed by putting it in the LHR group. However, because the surgical method itself was open, it was excluded from our study.

Nonetheless, according to the reviewer's opinion, we performed further analysis to investigate whether a conversion group differs from other groups. Although the surgical time was longer in this group compared to that in the LHR group, there was no difference in other postoperative complications and POI. We have added a supplement table summarizing the analysis of the three groups and have summarized the results in the discussion section.

2. **Comment: In Table 1, I see the data of the previous surgery (Hartmann method) but this data is not presented in Table 2. This is very important information to consider the result of matching and I highly recommend to include it.**

→ In the group that underwent open Hartmann's reversal, remarkably few cases were present where the previous operation was laparoscopic Hartmann's operation (3.9%); thus, we excluded Hartmann's method from matching in PSM and included this as a limitation in the discussion section. However, according to the reviewer's opinion, we have added the Hartmann method to Table 2 as well.

3. **Comment: Hartmann operation will be considered when the anastomosis is considered**

to be difficult. However, reason of this decision will be various kinds of things; for example, perforation, severe comorbidity, elderly patients, proficiency of the surgeon, etc. It would be better to include more specifc data why the patients underwent Hartmann operation at the beginning.

→ Our study focused on Hartmann's reversal and investigated data from multi-institutional electric medical records. Furthermore, there were cases where Hartmann's operation was performed at institutions other than our six institutions, and there were no records from the other hospital; even though there was a surgical record for Hartmann's operation, there was no mention of the reason why Hartmann's operation was performed, and we had to exclude that item. Therefore, we have added this as a limitation of our study in the discussion section.

4. **Comment: As the author mentioned in the limitation, there will be a selection bias since the patients with advanced cancer cannot undergo Hartmann reversal when they are under chemotherapy. It might be better to show how many patients underwent Hartmann surgery and how many had Hartmann reveersal.**

→ According to the reviewer's opinion, the number of patients who underwent Hartmann's surgery in our institutes was investigated. In total, 1076 patients underwent Hartmann's operation in our hospital, and among them, 306 patients underwent Hartmann reversal. The remaining 31 patients who underwent Hartmann reversal in our institutes were patients who had undergone Hartmann's operation at other hospitals. This has been added to Figure 1.

5. **Comment: The frequency of POI seems to be quite different. Is there any difference in the required treatment for these patient comparing LHR and OHR group? Were there any case which needed another surgery to treat POI? The primary outcome was set as POI and I recommend the author to analyze more deeply in it.**

→ In the LHR group, two POI patients showed an improvement in symptoms at 2 and 3 days, respectively, after L-tube insertion with parenteral nutritional support. However, one patient underwent laparoscopic adhesiolysis with transverse colectomy to resolve adhesions.

Of 13 POI patients in the OHR group, three patients showed spontaneous improvement in their symptoms without L-tube insertion. However, one patient underwent open adhesiolysis with T-colostomy to resolve the adhesion.

We have added these in the results section.

6. **Comment: Although there was no significant difference, I am quite surprised that the incisional hernia was more frequent in LHR group. Why do you think this happened?**

→ The exact cause is not known, but the occurrence of incisional hernia was confirmed by CT; since CT was performed in only about 38 out of 63 patients in each group, this may have resulted in a difference in incidence rates.
