# Supplementary material for: Postoperative effects of laparoscopic Hartmann reversal: A multicenter propensity score matched study
Source: PLoS One. 2023 Jun 2;18(6):e0286562. doi: 10.1371/journal.pone.0286562 (PMC10237654; doi:10.1371/journal.pone.0286562)
Supplement: Attachment — Submitted filename: Comment PLOS One - PONE-D--23-05434.docx [file pone.0286562.s006.docx]

**Comment PLOS One - PONE-D--23-05434**

**Title**: Postoperative effects of laparoscopic Hartmann reversal: A multicenter propensity

score matched study.

The title is in line with study objectives and the results

**Abstract:** Structured and well written

**Background**: Detailed information on statement of problem as well as rational for the study clearly presented.

**Methods:** Fairly well described.

- Study design should be mentioned even though it is apparent from the methodology.
- Minimum sample size for the study was not calculated. This is desirable, as this will show whether the sample size in this study meets the minimum calculated sample size. If the sample size used is less than the calculated minimum sample size, then this can be a limitation in the study.

**Result:** Well written in details with relevant tables and figures

- The first column for most of the tables are without a label
- The state number of days for POI, hospital stay, etc are not really clear. Are these mean values or median? If it is mean value, please indicate it in the sentence and provide the standard deviation (SD) as well.
- Line 126 - Frequency cannot be in percentage. Please correct it. Is it the prevalence?

**Discussion:** The study findings are well discussed, with study limitations provided.

**Conclusion:** Clearly written with appropriate recommendation.
