# Supplementary material for: Postoperative effects of laparoscopic Hartmann reversal: A multicenter propensity score matched study
Source: PLoS One. 2023 Jun 2;18(6):e0286562. doi: 10.1371/journal.pone.0286562 (PMC10237654; doi:10.1371/journal.pone.0286562)
Supplement: Attachment — Submitted filename: Response_to_Reviewers.docx [file pone.0286562.s009.docx]

***Review Comments to the Author***

**Reviewer #1: Authors have addressed all the issues and suggestions raised in the earlier review quite satisfactorily.**

- We thank the reviewer for reviewing the revised manuscript.

**Reviewer #2: Thank you for the correction. Most of my comments are fully addressed. One last comment from me is below.**

**1. I recommend the author to exclude the details for incisional hernia. Author have pointed out that almost half of patients did not have CT evaluation. Considering this fact, this result seems to be missleading.**

- We thank the reviewer for the considerate comments. Accordingly, we have excluded the details of incisional hernia from the revised manuscript.
